# Quantification of the internalization patterns of superparamagnetic iron oxide nanoparticles with opposite charge

**DOI:** 10.1186/1477-3155-10-28

**Published:** 2012-07-10

**Authors:** Christoph Schweiger, Raimo Hartmann, Feng Zhang, Wolfgang J Parak, Thomas H Kissel, Pilar Rivera_Gil

**Affiliations:** 1 Pharmaceutics and Biopharmacy, Faculty of Pharmacy, Philipps University of Marburg, Ketzerbach 63, Marburg, D 35037, Germany; 2 Biophotonics Group and WZMW, Institute of Physics, Philipps University of Marburg, Renthof 7, Marburg, D 35037, Germany

**Keywords:** Superparamagnetic iron oxide nanoparticles (SPIONs), Intracellular distribution, Charge, Coating, Size, Quantitative correlation analysis, Colocalization

## Abstract

Time-resolved quantitative colocalization analysis is a method based on confocal fluorescence microscopy allowing for a sophisticated characterization of nanomaterials with respect to their intracellular trafficking. This technique was applied to relate the internalization patterns of nanoparticles *i*.*e*. superparamagnetic iron oxide nanoparticles with distinct physicochemical characteristics with their uptake mechanism, rate and intracellular fate.

The physicochemical characterization of the nanoparticles showed particles of approximately the same size and shape as well as similar magnetic properties, only differing in charge due to different surface coatings. Incubation of the cells with both nanoparticles resulted in strong differences in the internalization rate and in the intracellular localization depending on the charge. Quantitative and qualitative analysis of nanoparticles-organelle colocalization experiments revealed that positively charged particles were found to enter the cells faster using different endocytotic pathways than their negative counterparts. Nevertheless, both nanoparticles species were finally enriched inside lysosomal structures and their efficiency in agarose phantom relaxometry experiments was very similar.

This quantitative analysis demonstrates that charge is a key factor influencing the nanoparticle-cell interactions, specially their intracellular accumulation. Despite differences in their physicochemical properties and intracellular distribution, the efficiencies of both nanoparticles as MRI agents were not significantly different.

## Background

The interaction of nanomaterials with cells and tissues is a key factor when considering their translation into clinical applications. Especially an effective accumulation of nanoparticles (NPs) inside certain tissues is beneficial for a great number of applications, such as hyperthermia, contrast enhancement in magnetic resonance imaging, cell tracking or theranostics [1-7]. Apart from colloidal stability, which is essential to ensure reproducibility as well as to influence the amount of cellular loading and toxicity, the surface chemistry/properties of the NPs control their cellular interactions [8]. Predominantly size, shape and surface charge of NPs influence their cellular internalization and distribution and thus their effective performance.

The overall uptake rate of nanoparticulate objects and their respective pathway of internalization can be manipulated by surface charge [9-11]. In general, cationic NPs have been found to display excellent properties for tracking applications as they enter cells with higher effectiveness [12] due to the interaction with the negatively charged glycocalix [13]. However, a higher degree of toxicity is often associated with these systems [14-17]. Nevertheless, also negatively charged NPs are massively incorporated by cells. In this respect it has to be mentioned that charged NPs strongly interact with serum proteins to form a protein corona [18-21], whose formation also depends on the NP charge. The rate of NP uptake is important, as insufficient cellular accumulation of NPs *e*.*g*. magnetic NPs can lead to deficient usage for example as imaging probes [22].

Thus a precise knowledge of their internalization mechanisms, endosomal sorting and resulting intracellular pathways are crucial aspects governing their fate, efficiency or toxicity. So far most of the techniques employed to study NP-cells interactions are based on qualitative analysis; thus being prone to subjectivity or to errors in the interpretation of results.

Typically intracellular trafficking is studied using fluorescence microscopy. By comparing the fluorescent pattern of labeled and internalized NPs with the distribution of cellular organelles possible intracellular pathways can be derived for the material. Following endocytic uptake, NPs are generally trapped in vesicular compartments. The detection and imaging of typical proteins associated to those enclosed structures allows their identification and allocation in for example endosomes or lysosomes. If such image material is super-imposed with signal gained for example from labeled NPs, structures associated with NP uptake, transport and processing can be identified. To analyze the uptake and enrichment of NPs inside a certain organelle fluorescent labeling of both, the nanomaterial and the organelle is typically performed. The uptake study is based on the correlation of the emission of the labeled nanomaterial with fluorescence signal of the organelle. If both structures are colocalizing within the detection volume the overlay of the corresponding two fluorescence image channels (for example red and green) would result in a new color value (yellow). In a qualitative manner the degree of colocalization can be estimated by looking at the super-imposed image. As a matter of fact, any processing having impact on the image’s histogram is influencing the “amount of yellow” in the overlay and altering the subjective impression of the degree of colocalization. For a sophisticated correlation of the image material of both structures, several approaches to perform a quantitative colocalization analysis exist. In intensity-based methods voxel or pixel intensities in both fluorescence channels are correlated by calculating for example Pearson’s or Manders’ colocalization coefficients [23,24]. In Li’s approach the correlation between the variations of the intensity-distributions within both channels are analyzed [25]. In object-based approaches the imaged structures are transformed into binary objects and the overlap is quantified [24]. In live-cell imaging also methods for trajectory correlation of those binary objects have been introduced [26]. Nevertheless, as long as single nanoparticle detection and tracking is hard to realize by conventional confocal microscopy the relevance of trajectory correlation is quite low, although the results seem to bear good prospects due to the discrimination of false colocalization caused by low axial resolution.

In order to validate our analysis methodology as well as to correlate differences in the physicochemistry of the NPs to different cellular behavior, the NPs were synthesized according to different protocols to produce NPs with different physicochemical properties. Especially surface chemistry and thus an opposite charge was selected on purpose, to influence the internalization rates of the NPs and thus proof our methodology. Due to the different synthetic protocols used, the colloidal stability and the size distribution of both NPs were also altered. According to their great potential in biomedical applications [6,7,27,28], superparamagnetic iron oxide NPs (SPIONs) were selected as systems to investigate NP internalization patterns; firstly qualitatively *via* flow cytometry (Fluorescence-Activated Cell Sorting, FACS) and Confocal Laser Scanning Microscopy (CLSM) and then by quantitative correlation analysis. Additionally, possible alterations in the relaxation times in A549 lung carcinoma cells were quantitatively evaluated.

## Results

Water-soluble SPIONs were synthesized either *via* aqueous coprecipitation [29] or *via* thermal decomposition of organometallic precursor molecules with subsequent phase transfer to aqueous solution [30-32]. Both methods lead to hydrophilic NPs suitable for biomedical applications.

The different synthesis strategies for formation of γ-Fe_2_O_3_ NPs clearly had an impact on the resulting NP morphology. Inorganic cores generated by aqueous co-precipitation following Massart’s protocol [29] were found to be inhomogenously spherically-shaped. Those coming from thermal decomposition of organometallic precursor molecules in organic solvent had homogenous, almost spherical shape and better size distribution. Analysis of the Fe_2_O_3_ core diameters (*i*.*e*. the inorganic Fe_2_O_3_ part without the organic surface coating) on transmission electron microscopy (TEM) micrographs revealed mean diameters of 10.4 ± 2.4 nm and 10.8 ± 0.12 nm for the synthesis performed in aqueous and organic solution, respectively (see Additional file 1: Figure SI-1.c in the SI). Adsorptive attachment of poly(ethylene imine) (PEI) to stabilize the NPs in solution completed the synthesis of positively charged γ-Fe_2_O_3_-PEI NPs. In contrast, hydrophobic interaction *via* intercalation of polymer (poly(isobutylene-alt-maleic anhydride), PMA) strands between surfactant alkyl chains formed the final step in producing hydrophilic negatively charged γ-Fe_2_O_3_-PMA NPs [33]. It is important to point out that coupling PEI to the γ-Fe_2_O_3_ NPs turned out to be essential to stabilize the NPs generated by aqueous co-precipitation in solution. The absence of PEI led to strong agglomeration, making some kind of characterization procedure of the NPs (cf. Additional file 1: Figure SI-1.f) difficult. Hydrodynamic diameters for the two polymer-modified formulations, γ-Fe_2_O_3_-PEI and γ-Fe_2_O_3_-PMA NPs as measured by dynamic light scattering in ultrapure water amounted to 16 ± 5 nm and 22 ± 7 nm, respectively (cf. Table 1). Both types of NP suspensions exhibited unimodal size distributions and zeta potentials of comparable absolute value, in numbers +53 ± 11 mV for γ-Fe_2_O_3_-PEI and −38 ± 6 mV for γ-Fe_2_O_3_-PMA (cf. Table 1). The impact of the preparation technology on magnetic features of the samples was investigated by monitoring the field-dependent magnetization with a SQUID (Superconducting QUantum Interference Device) system (cf. Table 1 and Additional file 1: Figure SI-2). All recorded curves showed lack of remanence and typical sigmoidal characteristics. The reader is referred to the SI ( Additional file 1: § SI-1 and § SI-2) for a detailed description of the synthesis and physicochemical characterization of both NP formulations.

**Table 1:** Physicochemical parameters of SPIONs as used in this work

	**hydrodynamic diameter[nm]**	**polydispersity index**	**zeta potential [mV]**	**saturation magnetization [emu g^-1^]**
γ-Fe_2_O_3_-PEI	16.2 ± 5.4	0.144 ± 0.019	+53.2 ± 11.0	23.7
γ-Fe_2_O_3_-PMA	22.1 ± 7.1	0.321 ± 0.025	−38.0 ± 5.6	16.4

**Figure 1:**
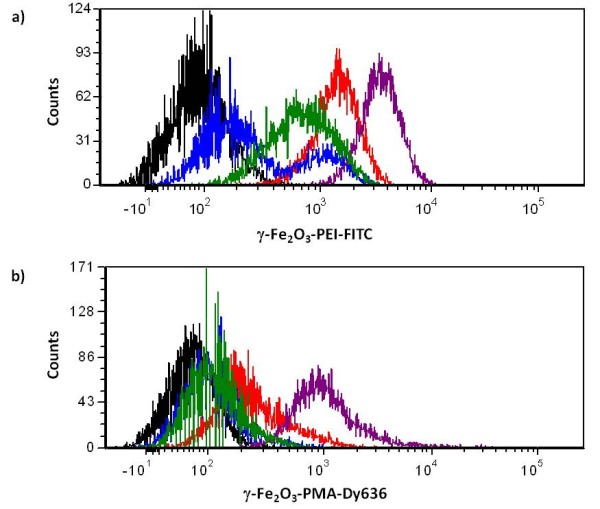
**Cellular uptake kinetics of nanoparticle formulations (a) γ-Fe**_**2**_**O**_**3**_**-PEI-FITC and (b) γ-Fe**_**2**_**O**_**3**_**-PMA-Dy636.** Cells were incubated with distinct amounts of the respective particle systems (1 μg/ml [Fe]) for time periods of 0 min (black line), 15 min (blue line), 60 min (green line), 4 h (red line), and 24 h (purple line). Fluorescence intensities were recorded by means of flow cytometry for a total of 10,000 events on channels FITC (excitation 488 nm) and APC-A (630 nm).

When incubating the lung carcinoma cell line A549 with fluorophore-bearing γ-Fe_2_O_3_ NPs, different uptake patterns were qualitatively observed for the two species. First of all it has to be remarked that due to a significant inferior colloidal stability of γ-Fe_2_O_3_-PEI-FITC NPs in growth medium (10 % serum-containing media) compared to γ-Fe_2_O_3_-PMA-Dy636 NPs it turned essential to establish a suitable exposure NP dose as well as the composition of the cell media, in which both NP systems had sufficient colloidal stability. An iron ([Fe]) concentration of 1 μg/ml in a 5 % serum-containing media turned out to be a good compromise between agglomeration, cell survival, and optical detection. Higher concentrations gave a better fluorescence signal (due to the fluorophores in the NP shell) but, in the case of γ-Fe_2_O_3_-PEI-FITC NPs suffered from strong agglomeration problems. NPs at lower concentrations were difficult to detect optically (cf. Additional file 1: Figure SI-6.a.i). It has to be pointed out that the concentrations are not absolutely comparable in terms of NPs per volume, as the mass comprises besides the inorganic Fe_2_O_3_ cores also the organic coating around their surface, which is different for both types of preparations. The quantity of serum proteins had to be lowered from 10 % (corresponding to the normal A549 growth media) to 5 % (cf. Additional file 1: Figure SI-6.a.ii and SI-6.a.iii). After having established the cell culture and NP incubation conditions the uptake of both formulations was studied with FACS and with CLSM. Positively γ-Fe_2_O_3_-PEI-FITC and negatively γ-Fe_2_O_3_-PMA-Dy636 charged NPs were internalized in a steady manner over the examined period of 24 hours. Nevertheless, the uptake of γ-Fe_2_O_3_-PEI-FITC NPs was taking place to an extent of about 40% within the first 4 h after incubation (cf. Figure 1). In contrast to that, γ-Fe_2_O_3_-PMA-Dy636 NPs were found to accumulate in cells only to a small extent within the first hours. The major fraction of these NPs was incorporated between time points 4 and 24 hours (cf. Figure 1), mostly after 8 h (cf. Additional file 1: Figure SI-6.b). Single-peaked mean fluorescence intensity signals indicated that there were no cell population subsets with lower degrees of NP incorporation. Incubation of the cells under the same circumstances as for FACS measurements and characterization by CLSM confirmed these results (cf. Additional file 1: Figure SI-6.b.i). Interestingly, negatively charged γ-Fe_2_O_3_-PMA-Dy636 NPs were faster incorporated by the cells in the presence of positively charged γ-Fe_2_O_3_-PEI-FITC NPs (cf. Additional file 1: Figure SI-6.b.i and SI-6.b.vi). These results suggest that upon concomitant incubation, complexes from positively and negatively charged NP were formed due to electrostatic interaction, finally leading to an increase in the uptake rate of the negatively charged NPs.

**Figure 2:**
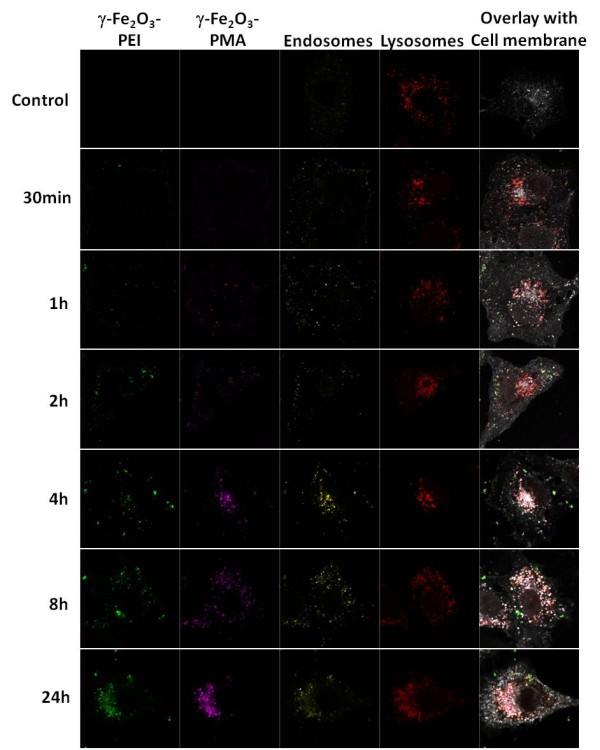
**Intracellular localization of SPIONs.** Cells were incubated concomitantly with both SPIONs (γ-Fe_2_O_3_-PEI-FITC and γ-Fe_2_O_3_-PMA-Dy636) at a final concentration of 1 μg/ml [Fe] for time periods of 30 min, 1 h, 2 h, 4 h, 8 h, and 24 h. Cells were then stained with wheat germ agglutinin, for EEA1 and for LAMP-1 to visualize the Plasma Membrane (white), the Endosomes (yellow) and the Lysosomes (red), respectively. Fluorescence images were recorded with a LSM. Additionally, the same experiments were performed with cells incubated with each NP system (see Additional file 1).

The impact of different charge and surface coating of both NP carriers on their intracellular pathways was analyzed by CLSM. For this purpose A549 cells, which were exposed to both NPs for different periods of time (individually or concomitantly) were stained for different organelles *i*.*e*. early endosomes, lysosomes, actin cytoskeleton and the plasma membrane (cf. Additional file 1: SI-6.b). Figure 2 shows the results of the intracellular localization of the NP complexes, whereby positively and negatively NPs were added simultaneously to the cells. In addition, the colocalization of each NP carrier with the different organelles upon time can be seen in Additional file 1: Figure SI-6.b. After 30 min the first fluorescent signal of γ-Fe_2_O_3_-PEI-FITC NPs was detectable. However the γ-Fe_2_O_3_-PMA-Dy636 NPs were firstly visualized after 60 min. Interestingly, at this early time points negatively charged γ-Fe_2_O_3_-PMA-Dy636 NPs clearly colocalized spatially with early endosomes near to the plasma membrane, whereas the positively charged counterparts, γ-Fe_2_O_3_-PEI-FITC NPs, were not found inside the endosomes. The endosomes migrate towards lysosomal structures wherein the first NPs were detectable after approximately 4–8 hours. After 24 h most of the NPs γ-Fe_2_O_3_-PEI-FITC as well as γ-Fe_2_O_3_-PMA-Dy636 were found inside the lysosomes. One can speculate that the absence of γ-Fe_2_O_3_-PEI-FITC NPs in the endosomes is due to the presence of PEI, which might manage to transfer the NPs out of the endosomes due to the proton-sponge effect [34]. In this case the NPs should be found free in the cytosol of the cell. To confirm this assumption, the actin cytoskeleton was stained and the possible colocalization of the free NPs was studied. As can be seen in Figure 2 (see also Additional file 1: Figure SI-6.b.vii), γ-Fe_2_O_3_-PEI-FITC NPs were not found at detectable level in the cytosol of the cells. As expected γ-Fe_2_O_3_-PMA-Dy636 NPs were also not found there but rather inside vesicular structures as their counterparts, γ-Fe_2_O_3_-PEI-FITC NPs did.

**Figure 3:**
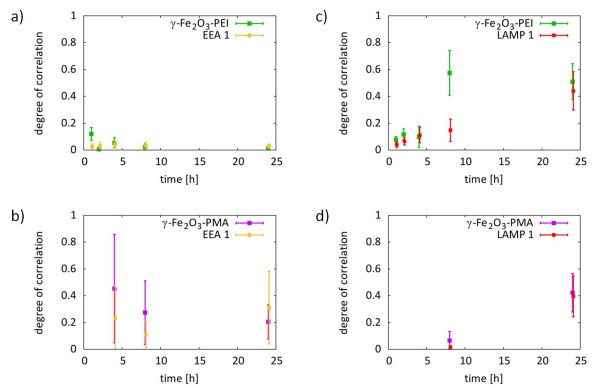
**Quantification of colocalization.** Manders’ coefficients M_1_ and M_2_ represent the correlation between the intracellular locations of γ-Fe_2_O_3_-PEI (green) and γ-Fe_2_O_3_-PMA (pink) with early endosomes (yellow) and lysosomes (red), respectively.

Colocalization studies *via* CLSM images without further data treatment are merely qualitative in nature so that different labeling efficiencies of the two NP systems as well as the different optical properties of the fluorophores conjugated to the NPs can induce erroneous interpretations. In order to get absolute comparability between the intracellular localization of γ-Fe_2_O_3_-PEI-FITC and γ-Fe_2_O_3_-PMA-Dy636 NPs a quantitative colocalization analysis of both NPs with the different organelles was performed upon time (cf. Methods and Supporting Information) [35]. As can be seen in Figure 3, the results confirmed the qualitative analysis by looking at the overlay of the different fluorescence channels (cf. Figure 2). Immediately after addition of the NPs to the cells, γ-Fe_2_O_3_-PEI-FITC NPs did not colocalize with the endosomes (cf. Figure 3.a) though in contrast γ-Fe_2_O_3_-PMA-Dy636 NPs did to some extend (cf. Figure 3.b). Approximately 45 % of all γ-Fe_2_O_3_-PMA-Dy636 NP signal was overlapping with the endosomes but only 22 % of endosome signal was overlapping with the NPs at 4 h incubation time. At later points of time both NP types were found in the lysosomes (cf. Figure 3.c and 3.d). A significant fraction of γ-Fe_2_O_3_-PEI-FITC NPs colocalized with lysosomal structures after 8 h incubation time, but quite few lysosomes contained NPs. Interestingly, the analysis suggests that at early incubation times some γ-Fe_2_O_3_-PEI-FITC NPs were already in the lysosomes. After 24 h incubation time, a large fraction of γ-Fe_2_O_3_-PEI-FITC and γ-Fe_2_O_3_-PMA-Dy636 NPs colocalized with the lysosomes and a large fraction of lysosomes were containing NPs of both nature.

**Figure 4:**
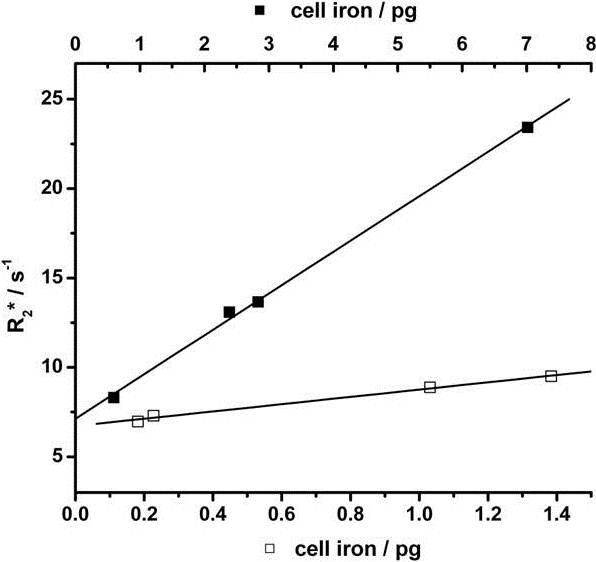
**Relaxation rates R**_2_* of agarose phantoms containing 10^**5**^**cells doped with SPIONs.** Data points represent intracellular iron levels after incubation with increasing amounts of γ-Fe_2_O_3_-PEI (▪) and γ-Fe_2_O_3_-PMA (□), respectively (1, 10, 30 and 50 μg [Fe]).

Finally, the impact of different charge and surface coatings of both NP carriers on their magnetic properties was studied. Relaxation parameters were gathered for agarose phantoms containing either freely dispersed NPs (γ-Fe_2_O_3_-PEI-FITC or γ-Fe_2_O_3_-PMA-Dy636) or cells loaded with certain amounts of SPIONs following incubation. Parameters of manufactured phantoms containing doped cells were dependent on the effective amounts of iron per cell. As expected, A549 incubation with high iron molarities caused non-proportional enhancement of intracellular accumulation. For γ-Fe_2_O_3_-PEI-FITC NPs, maximum incubation with a total of 100 μg [Fe] (as determined with ICP-OES) for instance led to intracellular iron levels of 6.9 pg per cell and subsequent relaxation rates R_2_* of 23.0 s^-1^, where R_2_* is indicative of absolute proton relaxation and signal darkening level. An identical application scheme of γ-Fe_2_O_3_-PMA-Dy636 NPs resulted in values of 1.4 pg per cell and R_2_* of 8.2 s^-1^. In comparison to that, relaxation rates R_2_* reached 140 s^-1^ and 134 s^-1^ for freely dispersed PEI-FITC and PMA-Dy636 NPs at equal “incubation” levels (data not shown). Despite the discrepancy in absolute R_2_* numbers, the efficiency of both SPION set-ups in reducing transverse relaxation times, often denoted as relaxivity r_2_*, turned out to be almost equivalent as derived from comparison of the slopes of the best-fit lines: 1.70 μM^-1^ s^-1^ and 1.72 μM^-1^ s^-1^ for freely dispersed PEI-FITC and PMA-Dy636 NPs (data not shown), 1.61 μM^-1^ s^-1^ and 1.58 μM^-1^ s^-1^ for cell containing PEI-FITC and PMA-Dy636 NPs, respectively (cf. Figure 4).

## Discussion

Several models are reported concerning the internalization of differently charged SPIONs [36]. However, only little efforts have been made so far to directly compare NP systems of equal dimensions and opposite charge with respect to their cellular uptake rate and intracellular fate. Especially a profound quantification of the colocalization of the NPs with different cellular structures is missing. Consequently, our approach consisted in eliminating size and shape as a key factor for NP uptake by keeping the dimensions of the two formulations constant. We hypothesized that, under these circumstances, the invasion into cells was predominantly dependent on the surface properties provided by the polymer coating, *i*.*e*. surface potential and the colloidal stability of incubated carriers.

Firstly, the synthesis strategy seems to affect the magnetic properties of the fabricated SPIONs. It is well-known that magnetization of inorganic colloids is determined primarily by their crystal diameter [37]. The results from TEM statistical analysis display a number-weighted and therefore one-dimensional quantity (cf. the TEM data in Additional file 1: Figure SI-1.c). As the magnetic moment of nanoparticles depends on their volume, the relative contribution of particles with larger size to the overall magnetization is higher. A mathematical approximation of a volume-weighted mean for both samples gave values of 11.5 nm each. Since mean diameters are virtually equal, we speculate that microstructural features of the magnetic cores are responsible for the different saturation magnetizations. On the one hand, the crystalline domains in the γ-Fe_2_O_3_-PMA NPs might be smaller than those in the γ-Fe_2_O_3_-PEI NPs. Another explanation for the differing M_sat_ values might be the existence of a magnetically dead layer on the maghemite surface which does not contribute to the collective magnetic moment of γ-Fe_2_O_3_ NPs. The general reduction in magnetization with respect to bulk maghemite can be attributed to several mechanisms such as spin canting or spin-glass-like behavior of the surface spins, both of them being effects which become more and more important with decreasing particle size [38,39]. Polymer shielding of the naked Fe_2_O_3_ cores induced further lowering of gram-standardized saturation magnetizations, which becomes logical as the organic material does not add to the magnetic properties of the respective particle systems. As already pointed out, direct mass-correlated comparison of both types of NPs is complicated due to the fact that they have different surface coatings and thus mass contributions of organic material. Moreover, organic ligands used to stabilize SPIONs might lead to quenching of surface magnetic moments [40]. The sigmoidal curves displayed in the SI ( Additional file 1: §2) are indicative for superparamagnetism of both γ-Fe_2_O_3_-PEI and γ-Fe_2_O_3_-PMA NPs. This feature is not only beneficial due to the availability of giant magnetic moments, but also due to the reduction in agglomeration tendency which is attributable to complete paramagnet-like loss of magnetization at zero external fields.

Secondly, differences in the charge of the NPs clearly affected their intracellular internalization route, rate and distribution. This statement was achieved by combination of the results obtained FACS and with CLSM followed by a time resolved quantitative analysis of the internalization patterns of both NP types. The conclusions drawn by eye-based interpretation of superimposed, fluorescent images presenting the distribution of NPs and certain cellular structures are strongly biased by any acquisition parameters and image processing. For a first impression or a proof of principle this method may be sufficient but the generalization of any observation has to be not taken literally. The averaging over colocalization data of several individual experiments and the imaging of various cells for each data point is needed. For quantification, the described procedure of time-resolved colocalization analysis is a well suited tool that certainly helps to retrace NP internalization in a reproducible manner.

The saturable, steady, but non-linear uptake pattern of positively charged γ-Fe_2_O_3_-PEI NPs strongly suggests adsorptive endocytosis as the main mechanism of cell uptake. This is supported by the fact that electrostatic interaction between the positively charged NPs and the negatively charged glycocalix certainly favors fast attachment to the cell membrane and subsequent ingestion of cationic species. It also goes along with the fact that the colloidal stability of the γ-Fe_2_O_3_-PEI NPs is limited. Furthermore, it has to be noted that positively charged NPs have been reported to interact differently with serum proteins at physiological pH than negatively charged NPs [21]. Different protein coronas are likely to influence cellular uptake. Finally, from the CLSM images of the cells incubated with positive γ-Fe_2_O_3_-PEI NPs over time and over different concentrations (cf. Additional file 1: Figure SI-6) it can be observed that accumulation of the positively charged NPs in the extracellular side of the cell membrane plays a key role, not only on the adsorption to the cell membrane (as commented before) but also on the fast rate of internalization. It appears that uptake of the positively charged NPs occurred only after membrane accumulation of the NPs, thus directly related to a specific NP concentration. These results suggested that there might be a concentration threshold responsible for the fast internalization as it happens for zwitterionic NPs [41]. Above the threshold NPs are rapidly internalized, however below this threshold NPs should be less efficiently internalized. Thus special attention should be paid to this insight. On the other hand, negatively charged γ-Fe_2_O_3_-PMA NPs seem to follow common endocytic internalization processes and even receptor-mediated endocytosis cannot be excluded. The uptake was steady and constant over time and more important, it was independent from membrane accumulation, thus excluding unnecessary thresholds. Furthermore, the negatively charged NPs strongly interact with serum proteins leading to the formation of a protein corona around the surface of the NPs [19-21]. Many of these proteins have specific ways of cellular entry. For example transferrin, which has been demonstrated to adsorb to the surface of negatively charged PMA-coated NPs [42] is well known to internalize *via* receptor-mediated endocytosis [43]. In this way, the uptake of these NPs may be controlled by the protein corona.

Sufficient and fast loading of certain cell types with SPIONs is for example desired for tracking purposes *via* magnetic resonance imaging [44]. The reason for this is the concentration-dependent enhancement of transverse proton relaxation in the vicinity of areas containing magnetic iron oxide NPs, thus leading to quick fading of MR signals and gain of contrast in T_2_-weighted images [45]. Based on the quantification of the intracellular iron concentration *via* ICP-OES (cf. Additional file 1: Figure SI-5), γ-Fe_2_O_3_-PEI NPs accumulate to a higher degree inside the cells compared to the negative γ-Fe_2_O_3_-PMA NPs. Thus γ-Fe_2_O_3_-PEI NPs should perform better than their anionic counterpart when being used for cell tracking tasks. However, it has to be pointed out that despite an optimization of the cell culture media and NP dose (*i*.*e*. reduced serum quantity as well as adequate NP dose) to increase the stability of the γ-Fe_2_O_3_-PEI-FITC NPs, agglomeration was still observed for this formulation and the agglomerates were strongly attached to the cell membrane (cf. Additional file 1: Figure SI-6.a.ii and SI-6.a.iii). Trypsinization of the cells did not remove the agglomerates of γ-Fe_2_O_3_-PEI-FITC NPs attached to the cell membrane and thus signal could also result from γ-Fe_2_O_3_-PEI-FITC NPs only adherent to the outer cell membrane.

Finally, as an alternative way for probing the efficiency of both types of NPs as contrast agents for MRI, agarose phantoms containing NP-labeled cancer cells were subjected to MR measurement sequences. Phantom matrices act as versatile human tissue equivalents, as alteration of their basic composition allows for the imitation of specific intracorporal regions and appendant relaxation properties [46]. Most effective signal darkening in T_2_-weighted MRI maps, denoted as high absolute relaxation rate values R_2_*, was observed for freely dispersed NPs and, to a lower extent, cell dispersions carrying large amounts of γ-Fe_2_O_3_-PEI NPs. As a more reliant measure of proton relaxation yield/efficiency, transversal relaxivity r_2_* values were calculated by normalization of the results to the iron concentration. The differences in relaxivity r_2_* between freely dispersed NPs and cell-confined NPs were relatively small and were supposed to result from less efficient proton spin interaction of magnetic NPs upon entrapment inside cells or cell organelles. Thus, besides concentration of magnetic materials as the main factor for signal improvement, intracellular confinement plays a second, yet subordinate role in this context and has an impact on the detected proton relaxation times [47]. Coming back to the magnetization properties of the tested NP formulations, we predicted higher molar relaxivities for the system γ-Fe_2_O_3_-PEI due to enhanced magnetic interactions with surrounding proton spins. Surprisingly, the efficiencies of both tested NP formulations were found to be in the same range.

## Conclusions

The physicochemical properties of the generated NPs (mainly charge and colloidal stability) were found to be a key factor governing the internalization into cells. The internalization patterns (i.e. uptake rates and intracellular localization) of SPIONs synthesized either directly in water or in organic solutions and with opposite charged, were completely different. The use of qualitative techniques like FACS and CLSM give interesting initial information in this regard however, a quantitative analysis is crucial to make statistically relevant conclusions. By real time quantitative correlation analysis the kinetic of NP internalization could be elucidated. Negatively charged SPIONs were found firstly in endosomes and lately in lysosomes whereas positively charged SPIONs were found exclusively inside lysosomes. Interestingly, not all the involved vesicles were found to be colocalizing with the NPs all over the time. Thus, elucidating dynamics in NPs trafficking inside the cells depending on their charge.

## Methods

For a detailed description of the experimental procedure as well as for other additional experiments, the reader is referred to the supporting information ( Additional file 1).

### Nanoparticle synthesis

γ-Fe_2_O_3_ NPs were prepared following standard protocols since the acquirement of exact information about the crystalline structures of these kind of NPs is very controversial [48]. γ-Fe_2_O_3_ NPs were synthesized either *via* aqueous coprecipitation, according to the Massart protocol [29] or *via* thermal decomposition of organometallic precursor molecules following a published protocol by Hyeon and co-workers [30].

### Physicochemical characterization

Hydrodynamic diameters and ζ-potentials of hydrophilic NPs after polymer functionalization were assessed by dynamic light scattering (DLS). For magnetization studies, the lyophilized NP materials were placed into a Magnetic Property Measurement System MPMS® equipped with a 5 T magnet (Quantum Design, San Diego, CA) using superconducting quantum interference device (SQUID) technology.

### Cell culture and uptake studies

The human lung adenocarcinoma cell line A549 was maintained in Dulbecco’s Modified Eagle Medium (DMEM) supplemented with 10 % serum. The uptake kinetics was analyzed with (1) flow cytometry and (2) CLSM. For (1), some cells were incubated with either γ-Fe_2_O_3_-PEI-FITC or with γ-Fe_2_O_3_-PMA-Dy636 NPs at fixed iron concentrations (1 μg/ml). The concentration of iron [Fe] was measured by ICP-OES (inductively coupled plasma - optical emission spectroscopy) (see Additional file 1: §1.g). Following determined incubation times (0 min, 15 min, 60 min, 4 h, 24 h), cells were analyzed with respect to their fluorescent intensity *via* FACS, using a FACSCanto II (BD Biosciences, San Jose, CA). For (2), the cells were incubated with each NP system as well as with both NP systems concomitantly ( Additional file 1: Figure S6.b). Each NP species was diluted to a final iron concentration of 1 μg/ml and again the cells were incubated for different periods of time (30 min, 1 h, 2 h, 4 h, 8 h and 24 h). Cells were prepared for labeling as described in the supporting information. The cell membrane was stained with fluorescent wheat germ agglutinin and actin was colored applying fluorescent phalloidin (results are presented in the SI, Additional file 1: Figure SI-6). To visualize the metabolic pathways of the NPs, immunostainings of lysosomal structures and early endosomes were performed. Lysosomes were stained using monoclonal mouse anti-human LAMP1/CD107a antibodies (Developmental Studies Hybridoma Bank), while early endosomes were labeled with polyclonal rabbit anti-human EEA1 immunoglobulin (Cell Signaling). To excite and collect all fluorescence markers *i*.*e*. both types of NPs, cell membrane, actin cytoskeleton, lysosome and endosome simultaneously, the secondary antibodies used for the cellular structures had to be carefully chosen to minimize crosstalk, especially between the NPs and the cell membrane. Therefore the dyes conjugated to the antibodies were selected to absorb in the UV region of the spectra. In detail donkey anti-mouse DyLight405-ABs (Jackson ImmunoResearch) were used at 1 μg/ml to detect the LAMP1 specific primary antibodies while goat anti-rabbit AlexaFlour430 conjugated immunoglobulin (Invitrogen) was used as a secondary antibody for early endosomes at 30 μg/ml (both diluted in PBS containing 1 % BSA). For examination a LSM 510 Meta (Zeiss) microscope was used equipped with lasers emitting at 405, 488, 543 and 633 nm.

### Quantitative analysis of colocalization studies

The intracellular distributions of both nanoparticle species were correlated with the locations of early endosomes and lysosomes over time to study the intracellular trafficking of both systems (see Additional file 1: SI, §7). Therefore, A549 adenocarcinoma cells were incubated with either γ-Fe_2_O_3_-PEI-FITC or with γ-Fe_2_O_3_-PMA-Dy636 NPs at fixed iron concentrations (1 μg/ml) for different periods of time followed by an immunostaining of either early endosomes or lysosomes, performed as described above. For each of the combinations given in Additional file 1: SI, §7, Table 1 at least 20 cells were imaged using a highly corrected CLSM. The degree of colocalization of fluorescence signal originating from nanoparticles and labeled endosomes (EEA1) or lysosomes (LAMP1) was quantified by calculating Manders’ distinct colocalization coefficients M_1_ and M_2_ for the confocal image material: $M_{1}=\frac{\sum R_{i,coloc}}{\sum Ri}\in \left[0,1\right]$ and $M_{2}=\frac{\sum G_{i,coloc}}{\sum Gi}\in \left[0,1\right]$

R_i_ and G_i_ are the pixel intensities of pixel i in channel R (nanoparticles) and G (endosomes or lysosomes). “coloc” are pixels in which colocalization was observed. In our calculations M_1_ represents the degree of colocalization of fluorescence signal from one nanoparticle species with signal coming either from stained endosomes or lysosomes while M_2_ covers the situation with regard to the organelles. An image providing a high value of M_1_ but a low value for M_2_ can be interpreted as follows: Most of the detected particles are present in the particular cellular compartments but the largest fraction of these organelles is not including nanoparticles anyhow.

### Agarose phantom relaxometry

A549 cells were plated at a density of 100,000 cells per well and were incubated with suspensions of SPIONs of different types γ-Fe_2_O_3_-PEI and γ-Fe_2_O_3_-PMA) and concentrations (1, 10, 30 and 50 μg/ml) for 24 hours. After PBS washing and trypsinization, cell numbers were counted using a Neubauer chamber. Quantification of cell-internalized iron was realized by ICP-OES after cell lysis in concentrated nitric acid (600 μl) for 4 hours. Phantoms for MR relaxometry were produced by dispersing 10^5^ SPION-doped A549 cells in agarose (1 % w/v). Magnetic resonance (MR) imaging studies concerning the T_2_ and T_2_* relaxation times of the respective phantoms were carried out on a 7 T Bruker ClinScan 70/30 USR (Bruker BioSpin, Rheinstetten, Germany). For measurements of transverse T_2_ relaxation times, spin-echo multicontrast sequences were run at T_R_ values of 2000 ms, varying spin echo times T_E_ (10–120 ms with an increment of 10 ms), field of view 75x75 mm, matrix 128x128 and slice thickness 0.6 mm. Data quantification was achieved by evaluating such created DICOM images. Relaxation times T_2_ could be derived by analyzing regions of interest (ROI) within T_2_ maps generated by the overlay of successive spin-echo images, using a monoexponential fitting of the signal intensity (I) decay curve: I(t) = I_0_exp(−t/T_2_), where I_0_ is the signal magnitude at equilibrium and t the particular echo time. Effective transverse relaxation times (T_2_*) were calculated from T_2_*-weighted images taken with the following settings: gradient-echo multicontrast with T_R_ = 350 ms, multiple spin echo times T_E_ (3–32 ms), field of view 89x89 mm, matrix 128x128, slice thickness 0.5 mm. T_2_* values were obtained correspondingly by fitting the MRI signal intensities of the acquired maps versus echo times T_E_.

## Misc

Christoph Schweiger and Raimo Hartmann contributed equally to the realization of this work.

## Competing interests

We (the authors) wish to confirm that there are no known conflicts of interest associated with this publication and there has been no significant financial support for this work that could have influenced its outcome.

## Authors’ contributions

The concept was designed by WJP, THK, and PRG. Experiments and data analysis were performed by CS, RH, FZ, and PRG. All authors read and approved the final manuscript.

## Authors’ information

Dr. C. Schweiger studied Pharmacy and obtained his PhD under the supervision of Prof. Dr. T. H. Kissel in the Department of Pharmaceutical technology in the Philipps University of Marburg.

R. Hartmann studied Physics at the University of Marburg and is currently doing his PhD under the supervision of Dr. P. Rivera Gil and Prof. Dr. W. J. Parak.

Dr. F. Zhang received his bachelor degree in 2000 from Biology School of Inner Mongolia University and his Ph. D degree in 2006 from Shanghai Institute of Applied Physics, Chinese Academy of Sciences. After a postdoctoral stay in the group of Prof. Dr. W. J. Parak in the University of Marburg, Dr Zhang moved as a senior research fellow to Washington University (Bioengineering Dep.). He is currently employed as a professor and a Ph.D advisor in Biology School in Inner Mongolia Agricultural University.

Prof. Dr. W. J. Parak obtained his PhD in biophysics at the Ludwig Maximilians Universität München, Germany in 1999 in the group of Prof. Dr. Hermann Gaub. After a postdoctoral stay at the University of California, Berkeley, CA, USA in the group of Prof. Dr. Paul Alivisatos he returned 2002 to Munich as Assistant Professor. Since 2007 he is Full Professor at the Physics Department of the Philipps Universität Marburg, Germany.

Prof. Dr. T. H. Kissel is currently retired. Until August 2012, he was Professor of Pharmaceutics & Biopharmacy and Department Head at Philipps-Universität Marburg, Germany, where he has been since 1991. He received his B.S. (Pharmacy) from Freiburg University (1971), his M. S. (Chemistry, 1974) and his Ph.D. (Medicinal Chemistry, 1976) from Marburg University.

Dr. P. Rivera Gil studied Pharmacy in Spain and obtained her PhD in Pharmacology at the Free University Berlin. She is currently a senior researcher in the group of Prof. Dr. W. J. Parak.

## Supplementary Material

Additional file 1 Supporting Information. Click here for file

## References

[B1] Jordan A Scholz R Wust P Fahling H Felix R Magnetic fluid hyperthermia (MFH): cancer treatment with AC magnetic field induced excitation of biocompatible superparamagnetic nanoparticles J Magn Magn Mater 1999 201 413 419 10.1016/S0304-8853(99)00088-8

[B2] Gonzales M Krishnan KM Synthesis of magnetoliposomes with monodisperse iron oxide nanocrystal cores for hyperthermia Journal of Magnetism and Magnetic Materials 2005 293 265 10.1016/j.jmmm.2005.02.020

[B3] Nasongkla N Bey E Ren JM Ai H Khemtong C Guthi JS Chin SF Sherry AD Boothman DA Gao JM Multifunctional polymeric micelles as cancer-targeted, MRI-ultrasensitive drug delivery systems Nano Letters 2006 6 2427 2430 10.1021/nl061412u 17090068

[B4] Huh YM Jun YW Song HT Kim S Choi JS Lee JH Yoon S Kim KS Shin JS Suh JS Cheon J In vivo magnetic resonance detection of cancer by using multifunctional magnetic nanocrystals Journal Of The American Chemical Society 2005 127 12387 12391 10.1021/ja052337c 16131220

[B5] Rivera_Gil P Yang F Thomas H Li L Terfort A Parak WJ Development of an assay based on cell counting with quantum dot labels for comparing cell adhesion within cocultures Nano Today 2011 6 20 27 10.1016/j.nantod.2010.12.006

[B6] Jenkins SI Pickard MR Granger N Chari DM Magnetic nanoparticle-mediated gene transfer to oligodendrocyte precursor cell transplant populations is enhanced by magnetofection strategies ACS Nano 2011 5 6527 6538 10.1021/nn2018717 21721568

[B7] Cho HS Dong Z Pauletti GM Zhang J Xu H Gu H Wang L Ewing RC Huth C Wang F Shi D Fluorescent, superparamagnetic nanospheres for drug storage, targeting, and imaging: a multifunctional nanocarrier system for cancer diagnosis and treatment ACS Nano 2010 4 5398 5404 10.1021/nn101000e 20707381

[B8] Mailander V Landfester K Interaction of Nanoparticles with Cells Biomacromolecules 2009 10 2379 2400 10.1021/bm900266r 19637907

[B9] Harush-Frenkel O Rozentur E Benita S Altschuler Y Surface charge of nanoparticles determines their endocytic and transcytotic pathway in polarized MDCK cells Biomacromolecules 2008 9 435 443 10.1021/bm700535p 18189360

[B10] Chung YI Kim JC Kim YH Tae G Lee SY Kim K Kwon IC The effect of surface functionalization of PLGA nanoparticles by heparin- or chitosan-conjugated Pluronic on tumor targeting Journal Of Controlled Release 143 374 382 10.1016/j.jconrel.2010.01.017 20109508

[B11] Ge YQ Zhang Y Xia JG Ma M He SY Nie F Gu N Effect of surface charge and agglomerate degree of magnetic iron oxide nanoparticles on KB cellular uptake in vitro Colloids And Surfaces B-Biointerfaces 2009 73 294 301 10.1016/j.colsurfb.2009.05.031 19564099

[B12] Villanueva A Canete M Roca AG Calero M Veintemillas-Verdaguer S Serna CJ Morales MD Miranda R The influence of surface functionalization on the enhanced internalization of magnetic nanoparticles in cancer cells Nanotechnology 2009 20 10.1088/0957-4484/20/11/115103 19420433

[B13] Martin AL Bernas LM Rutt BK Foster PJ Gillies ER Enhanced Cell Uptake of Superparamagnetic Iron Oxide Nanoparticles Functionalized with Dendritic Guanidines Bioconjugate Chemistry 2008 19 2375 2384 10.1021/bc800209u 19053308

[B14] Luo JT Xiao K Li YP Lee JS Xiao WW Gonik AM Agarwal RG Lam KS The effect of surface charge on in vivo biodistribution of PEG-oligocholic acid based micellar nanoparticles Biomaterials 2011 32 3435 3446 10.1016/j.biomaterials.2011.01.021 21295849 PMC3055170

[B15] Xia T Kovochich M Liong M Zink JI Nel AE Cationic polystyrene nanosphere toxicity depends on cell-specific endocytic and mitochondrial injury pathways ACS Nano 2008 2 85 96 10.1021/nn700256c 19206551

[B16] Breunig M Lungwitz U Klar J Kurtz A Blunk T Goepferich A Polyplexes of polyethylenimine and per-N-methylated polyethylenimine-cytotoxicity and transfection efficiency Journal Of Nanoscience And Nanotechnology 2004 4 512 520 10.1166/jnn.2004.080 15503437

[B17] Petersen H Fechner PM Martin AL Kunath K Stolnik S Roberts CJ Fischer D Davies MC Kissel T Polyethylenimine-graft-poly(ethylene glycol) copolymers: Influence of copolymer block structure on DNA complexation and biological activities as gene delivery system Bioconjugate Chemistry 2002 13 845 854 10.1021/bc025529v 12121141

[B18] Walczyk D Bombelli FB Monopoli MP Lynch I Dawson KA What the Cell "Sees" in Bionanoscience Journal of the American Chemical Society 2010 132 5761 5768 10.1021/ja910675v 20356039

[B19] Röcker C Pötzl M Zhang F Parak WJ Nienhaus GU A Quantitative Fluorescence Study of Protein Monolayer Formation on Colloidal Nanoparticles Nature Nanotechnology 2009 4 577 580 10.1038/nnano.2009.195 19734930

[B20] Cedervall T Lynch I Lindman S Berggård T Thulin E Nilsson H Dawson KA Linse S Understanding the nanoparticle–protein corona using methods to quantify exchange rates and affinities of proteins for nanoparticles Proceedings of the National Academy of Sciences of the United States of America 2007 104 2050 2055 10.1073/pnas.0608582104 17267609 PMC1892985

[B21] Lundqvist M Stigler J Elia G Lynch I Cedervall T Dawson KA Nanoparticle size and surface properties determine the protein corona with possible implications for biological impacts Proceedings of the National Academy of Sciences of the United States of America 2008 105 14265 14270 10.1073/pnas.0805135105 18809927 PMC2567179

[B22] Huang HC Chang PY Chang K Chen CY Lin CW Chen JH Mou CY Chang ZF Chang FH Formulation of novel lipid-coated magnetic nanoparticles as the probe for in vivo imaging Journal Of Biomedical Science 2009 16 86 10.1186/1423-0127-16-86 19772552 PMC2758848

[B23] Gonzalez RC Woods RE Digital Image Processing 2008 3 Prentice-Hall, Upper Saddle River, NJ

[B24] Manders EMM Verbeek FJ Aten JA Measurement Of Colocalization Of Objects In Dual-Color Confocal Images Journal Of Microscopy-Oxford 1993 169 375 382 10.1111/j.1365-2818.1993.tb03313.x 33930978

[B25] Li Q Lau A Morris TJ Guo L Fordyce CB Stanley EF A Syntaxin 1, Galpha(o), and N-Type Calcium Channel Complex at a Presynaptic Nerve Terminal: Analysis by Quantitative Immunocolocalization The Journal of Neuroscience 2004 24 4070 4081 10.1523/JNEUROSCI.0346-04.2004 15102922 PMC6729428

[B26] Vercauteren D Deschout H Remaut K Engbersen JFJ Jones AT Demeester J De Smedt SC Braeckmans K Dynamic Colocalization Microscopy To Characterize Intracellular Trafficking of Nanomedicines Acs Nano 2011 5 7874 7884 10.1021/nn2020858 21923168

[B27] Pankhurst QA Connolly J Jones SK Dobson J Applications of magnetic nanoparticles in biomedicine Journal Of Physics D-Applied Physics 2003 36 R167 R181 10.1088/0022-3727/36/13/201

[B28] Rivera_Gil P Hühn D del Mercato LL Sasse D Parak WJ Nanopharmacy: Inorganic nanoscale devices as vectors and active compounds Pharmacological Research 2010 62 115 125 10.1016/j.phrs.2010.01.009 20097288

[B29] Bee A Massart R Neveu S Synthesis of Very Fine Maghemite Particles Journal of Magnetism and Magnetic Materials 1995 149 6 9 10.1016/0304-8853(95)00317-7

[B30] Hyeon T Chemical synthesis of magnetic nanoparticles Chem Commun 2003 8 927 934 10.1039/b207789b 12744306

[B31] Casula MF Jun YW Zaziski DJ Chan EM Corrias A Alivisatos AP The Concept of Delayed Nucleation in Nanocrystal Growth Demonstrated for the Case of Iron Oxide Nanodisks Journal of the American Chemical Society 2006 128 1675 1682 10.1021/ja056139x 16448141

[B32] Casula MF Floris P Innocenti C Lascialfari A Marinone M Corti M Sperling RA Parak WJ Sangregorio C Magnetic Resonance Imaging Contrast Agents Based on Iron Oxide Superparamagnetic Ferrofluids Chemistry Of Materials 2010 22 1739 1748 10.1021/cm9031557

[B33] Lin C-AJ Sperling RA Li JK Yang T-Y Li P-Y Zanella M Chang WH Parak WJ Design of an amphiphilic polymer for nanoparticle coating and functionalization Small 2008 4 334 341 10.1002/smll.200700654 18273855

[B34] Akinc A Thomas M Klibanov AM Langer R Exploring polyethylenimine-mediated DNA transfection and the proton sponge hypothesis Journal of Gene Medicine 2005 7 657 663 15543529 10.1002/jgm.696

[B35] Zinchuk V Zinchuk O Okada T Quantitative Colocalization Analysis of Multicolor Confocal Immunofluorescence Microscopy Images: Pushing Pixels to Explore Biological Phenomena Acta Histochemica et Cytochemica 2007 40 101 10.1267/ahc.07002 17898874 PMC1993886

[B36] He C Hu Y Yin L Tang C Yin C Effects of particle size and surface charge on cellular uptake and biodistribution of polymeric nanoparticles Biomaterials 31 3657 3666 20138662 10.1016/j.biomaterials.2010.01.065

[B37] Kneller EF Luborsky FE Particle Size Dependence of Coercivity and Remanence of Single-Domain Particles Journal of Applied Physics 1963 34 656 10.1063/1.1729324

[B38] Lu AH Salabas EL Schuth F Magnetic nanoparticles: Synthesis, protection, functionalization, and application Angewandte Chemie-International Edition 2007 46 1222 1244 10.1002/anie.200602866 17278160

[B39] Fiorani D Testa AM Lucari F D'Orazio F Romero H Magnetic properties of maghemite nanoparticle systems: surface anisotropy and interparticle interaction effects Physica B-Condensed Matter 2002 320 122 126 10.1016/S0921-4526(02)00659-2

[B40] Paulus PM Bonnemann H van der Kraan AM Luis F Sinzig J de Jongh LJ Magnetic properties of nanosized transition metal colloids: the influence of noble metal coating European Physical Journal D 1999 9 501 504 10.1007/s100530050487

[B41] Jiang X Röcker C Hafner M Nienhaus GU Endo- and Exocytosis of Zwitterionic Quantum Dot Nanoparticles by Living Cells ACS Nano 4 6787 6797 21028844 10.1021/nn101277w

[B42] Jiang X Weise S Hafner M Röcker C Zhang F Parak WJ Nienhaus GU Quantitative Analysis of the Protein Corona on FePt Nanoparticles formed by Transferrin Binding J R Soc Interface 2010 7 S5 S13 10.1098/rsif.2009.0272.focus 19776149 PMC2843989

[B43] Karin M Mintz B Receptor-Mediated Endocytosis of Transferrin in Developmentally Totipotent Mouse Teratocarcinoma Stem-Cells Journal of Biological Chemistry 1981 256 3245 3252 6259157

[B44] Himmelreich U Dresselaers T Cell labeling and tracking for experimental models using Magnetic Resonance Imaging Methods 2009 48 112 124 10.1016/j.ymeth.2009.03.020 19362150

[B45] Laurent S Boutry S Mahieu I Vander Elst L Muller RN Iron Oxide Based MR Contrast Agents: from Chemistry to Cell Labeling Current Medicinal Chemistry 2009 16 4712 4727 10.2174/092986709789878256 19903138

[B46] Park SM Nyenhuis JA Smith CD Lim EJ Foster KS Baker KB Hrdlicka G Rezai AR Ruggieri P Sharan A Gelled versus nongelled phantom material for measurement of MRI-induced temperature increases with bioimplants Ieee Transactions on Magnetics 2003 39 3367 3371 10.1109/TMAG.2003.816259

[B47] Tanimoto A Oshio K Suematsu M Pouliquen D Stark DD Relaxation effects of clustered particles Journal of Magnetic Resonance Imaging 2001 14 72 77 10.1002/jmri.1153 11436217

[B48] Corrias A Mountjoy G Loche D Puntes V Falqui A Zanella M Parak WJ Casula MF Identifying Spinel Phases in Nearly Monodisperse Iron Oxide Colloidal Nanocrystal J Phys Chem C 2009 113 18667 18675 10.1021/jp9047677

