# Supplementary material for: Quantification of the internalization patterns of superparamagnetic iron oxide nanoparticles with opposite charge
Source: J Nanobiotechnology. 2012 Jul 10;10:28. doi: 10.1186/1477-3155-10-28 (PMC3431280; doi:10.1186/1477-3155-10-28)
Supplement: Additional file 1 — Supporting Information. [file 1477-3155-10-28-S1.pdf]

SUPPORTING INFORMATION

Quantification of the internalization patterns of superparamagnetic iron oxide nanoparticles with opposite charge

Christoph Schweiger[1,*], Raimo Hartmann[2,*], Feng Zhang[2], Wolfgang. J. Parak[2], Thomas Kissel[1], Pilar Rivera_Gil[2,#]

[1] Pharmaceutics and Biopharmacy, Faculty of Pharmacy, Philipps University of Marburg, Ketzerbach 63, 35037, Marburg, Germany

[2] Biophotonics Group and WZMW, Institute of Physics, Philipps University of Marburg, Renthof 7, 35037 Marburg, Germany

* Both authors have contributed equally to the realization of this work

[#] Corresponding author: pilar.riveragil@physik.uni-marburg.de

Outline

1 **Nanoparticle synthesis and characterization**

2 **Magnetization measurements**

3 **Kinetic of internalization via FACS**

4 **Cell culture and characterization via CLSM**

5 **Quantification of intracellular iron concentration**

6 **Kinetic of internalization and intracellular localization of SPIONs via CLSM: concentration and time dependence**

7 **Quantitative analysis of colocalization studies**

8 **References**

1 Nanoparticle synthesis and characterization

a) Synthesis in aqueous solution

γ-Fe_2O_3 nanoparticles (NPs) of the maghemite type were prepared via aqueous coprecipitation, according to the Massart protocol [1]. Briefly, ammonia solution (2.6 ml, 25 % w/v) was added to a slightly acidic solution of iron(III) chloride hexahydrate and iron(II)chloride tetrahydrate (100 ml, 0.13 M, molar ratio 2:1) (both purchased from Sigma-Aldrich) under constant stirring until persistence of a black slurry. After collection with a permanent magnet and triple washing with ultra pure water, particles were refluxed in a mixture of nitric acid (2 N HNO_3) and iron(III)nitrate nonahydrate (0.34 M) at 90 °C for 30 minutes in order to allow for complete oxidation of inorganic particles to the maghemite transformation γ-Fe_2O_3. The precipitate was gathered by magnetic decantation, and subsequently dispersed in 20 ml of water by vigorous shaking to yield a stable maghemite suspension. Following that, dispersions were reacted with poly(ethylene imine) (PEI) solution at a defined mass ratio of [Fe] to [PEI] of 1:2 under vigorous shaking for 30 minutes. Unbound polymer was removed by dialysis against a 100-fold excess of purified water, using Spectra/Por® membranes with MWCO 100,000 Da (Carl Roth, Karlsruhe, Germany). After 12 h of dialysis, external water was renewed in order to allow for depletive removal of the polymer.

b) Synthesis in organic solvents

SPIONs were synthesized using a published protocol by Hyeon and co-workers [2]. Briefly, 10 ml of octyl ether (Sigma) and 1.28 g of oleic acid (Sigma) were mixed and degassed in three-neck flask for 20 minutes at 60 °C. After 20 minutes temperature was increased to 100 °C. At this stage 0.28 ml of iron pentacarbonyl (Sigma) was injected and the temperature was increased up to refluxing temperature (~ 295-300 °C). The solution was kept at this temperature for 1 hour. During this time the initial yellow colour of the solution changed to black. After one hour the solution was cooled to room temperature and 0.34 g of dehydrated trimethylamine oxide (Sigma) was added. The temperature was increased to 130 °C. The solution was kept at this temperature for two hours. During this time the black colour of the solution changed into dark brown. After two hours the solution temperature was again increased to refluxing temperature in steps, each 15 °C/min. The solution was kept at refluxing temperature for another hour. During this time the solution colour again changed from dark brown to black. After one hour the reaction was stopped by removing the heating mantel. At room temperature 2-5 ml of toluene (Sigma) was added followed by 25-30 ml of methanol (Sigma). Methanol caused precipitation of the NPs, which were pelleted with centrifugation at a speed of 2800 rpm. The supernatant was removed and the precipitate was washed by using toluene and methanol. The precipitate containing the NPs was then redispersed in 10-20 ml of toluene.

c) Physicochemical characterization of SPIONs.

TEM characterization and size distribution

Size and morphology of hydrophilic iron oxide cores were investigated on a JEM-3010 transmission electron microscope (Jeol Germany, Eching, Germany) at an acceleration voltage of 300 kV. Maghemite suspension droplets were placed onto carbon-coated copper grids S160-3 (Plano, Wetzlar, Germany) and allowed to dry. Core dimensions were calculated by averaging at least 200 diameters registered by ImageJ software. The TEM data give the size of the inorganic iron oxide core, which is smaller than the hydrodynamic diameter as determined *via* DLS.

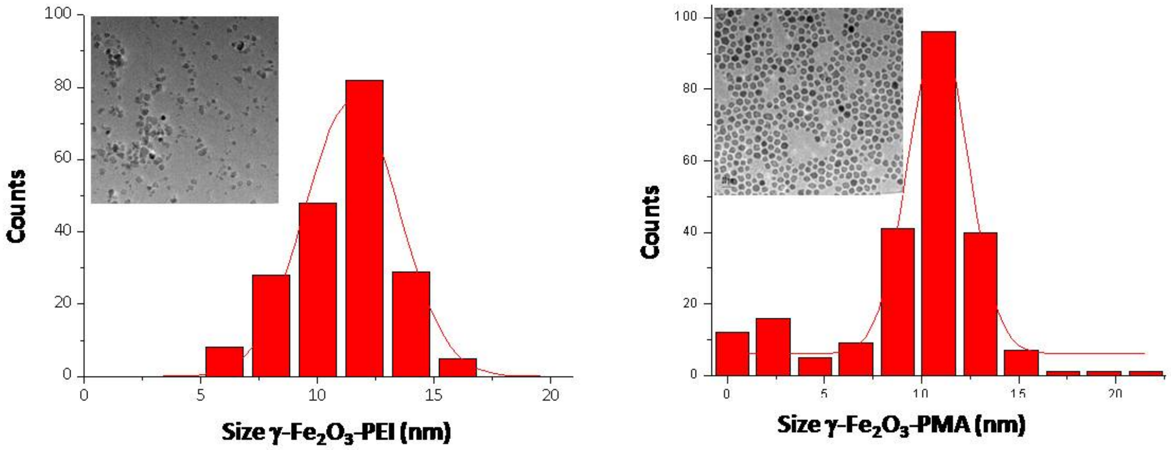

Figure SI-1.c.i – Morphological (left micrographs of each graphic) and size distribution of both SPIONs before coating.

ζ-potential

Hydrodynamic diameters and ζ-potentials of NPs after polymer functionalization were assessed by Dynamic Light Scattering (DLS) and Laser Doppler Anemometry (LDA), using a Zetasizer Nano ZS (Malvern Instruments, Herrenberg, Germany). DLS measurements were performed at 25 °C after appropriate dilution of the respective samples with ultra pure water, to avoid multiscattering events. As to LDA analysis, samples were dispersed in sodium chloride (10 mM) in order to maintain a constant ionic strength.

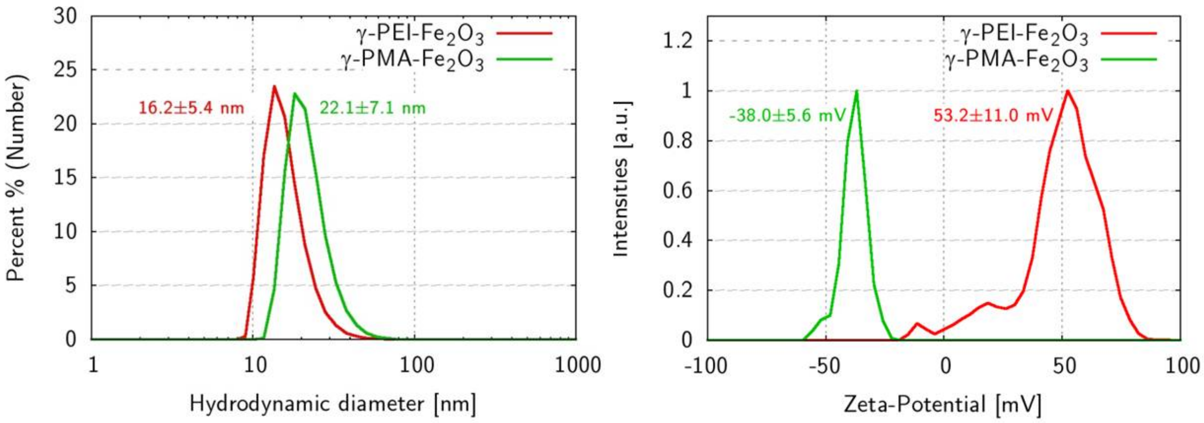

Figure SI-1.c.ii – Characterization of the hydrodynamic diameter and ζ-potentials of both SPIONs after coating.

d) Polymer coating and fluorophore conjugation

γ-Fe_2O_3-PEI-FITC

Dispersions were reacted with poly(ethylene imine) (PEI; gift from BASF) solution at a defined mass ratio of [Fe] to [PEI] of 1:2 under vigorous shaking for 30 minutes. Unbound polymer was removed by dialysis against a 100-fold excess of purified water, using Spectra/Por® membranes with MWCO 100,000 Da (Carl Roth, Karlsruhe, Germany). After 12 h of dialysis, external water was renewed in order to allow for depletive removal of the polymer. For fluorescent tagging of hydrophilic particles, green dye fluorescein isothiocyanate (FITC; Sigma-Aldrich) was conjugated to PEI 25k as previously described [3]. Briefly, PEI solution (1%, w/v) was mixed with FITC (10 mg/ml in dimethyl sulfoxide) in sodium carbonate buffer (1 M, pH 9.0) and incubated for 12 h at 4 °C. The mixture was transferred into an Amicon stirred cell (Millipore GmbH, Schwalbach, Germany), and unbound FITC was removed by repeted ultrafiltration through regenerated cellulose acetate membrane with MWCO 10 kDa. The labeled polymer was attached to naked maghemite NPs analogous to the synthesis protocol, thereby yielding γ-Fe_2O_3-PEI-FITC carriers in aqueous suspension.

γ-Fe_2O_3-PMA-Dy636

Amphiphilic polymer synthesis and the modification were according to previously published protocols [4-8]. Briefly, 2.70 g dodecylamine powder (≥98%, Fluka, #44170) was completely dissolved in 100 ml anhydrous tetrahydrofurane (THF, ≥99.9%, Aldrich, #186562) in a round flask. Then 3.084 g poly(isobutylene-alt-maleic anhydride) (PMA) powder (average Mw ~6,000 g/mol, Sigma) was added in and followed by heating to 55-60 °C for 1 hour under vigorous stirring. THF was then evaporated to

about 40 ml left and followed stirring again for overnight. Afterwards, the reaction mixture was completely dried by evaporation and the polymer was redissolved in 40 ml anhydrous chloroform (>=99%, Sigma) with a final monomer concentration of 0.5 M.

For the dye modified amphiphilic polymer, 1 mg amine containing dye (DY636, Dyomics Corp.) was firstly completely dissolved in 0.4 ml methanol, and then 512 µl above polymer solution was added and followed by overnight shaking incubation. The mixed solvents were exchanged to 25.6 ml anhydrous chloroform by evaporation of the old solvent followed by resuspension in chloroform. The final dye modified polymer concentration was 10 mM (monomer-motiv concentration). The dye modified amphiphilic polymer (PMA-Dy636) solution was then mixed with γ-Fe_2O_3 NPs by a molar ratio of 200 polymer motifs per nm^2 of NP's effective surface area[1]. The effective diameter d_{eff} of the NPs includes the hydrophobic surfactant shell around the inorganic cores[2] and the inorganic core diameter itself. After mixing, the solvent was slowly evaporated under reduced pressure until the sample was completely dried. The remaining solid film in the flask was re-dissolved in SBB12 (sodium borate, 50 mM, pH 12) under vigorous stirring until the solution turned clear. The PMA-Dy636 was then wrapped around the surface of the γ-Fe_2O_3 NPs in the buffer.

e) Optical characterization of SPION samples:

The optical properties (UV/vis absorption and steady-state fluorescence) of both purified NP samples were characterized by an Agilent 8450 spectrometer and a Fluorolog®-3 spectrometer, respectively. Both samples were diluted by buffers and the measurements were done with a quartz cuvette. For the emission spectra, 480 nm and 590 nm were used as excitation wavelength for γ-Fe_2O_3-PEI-FITC and γ-Fe_2O_3-PMA-Dy636, respectively.

---

[1]The core diameter of the Fe_2O_3 NPs was determined by the TEM analysis (*cf.* §1.c). The effective diameter d_{eff} includes the organic molecules which were considered to contribute a layer thickness of about 1 nm around the inorganic core. Then the surface area per NP A_{eff} was estimated by using the calculation equation of $A_{eff} = 4\pi \bullet (d_{eff}/2)^2$.

[2]This is the diameter before the polymer-coating. They were determined by adding 2 nm (assumed 1 nm thickness shell contributed by surfactants) to the inorganic core diameter determined by TEM analysis.

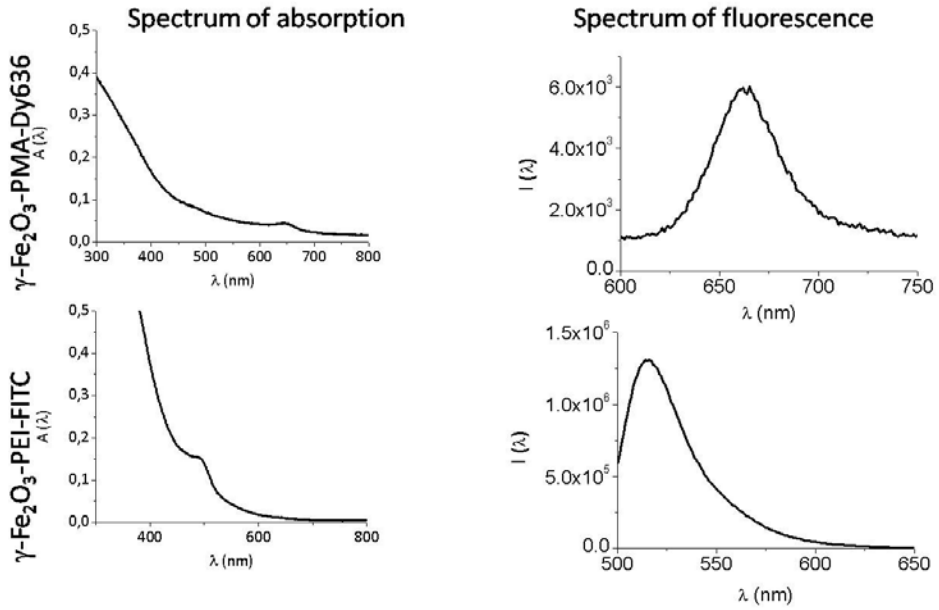

Figure SI-1.e – Absorption and emission spectra of both fluorescently-labeled SPIONs.

f) Purification of conjugated NPs

γ-Fe_2O_3-PEI-FITC

Purification of the aqueous γ-Fe_2O_3-PEI-FITC NP suspensions was performed by dialysis in several cycles against an excess of ultra pure water, using regenerated cellulose membranes with 100 kDa MWCO (SpectraPor®, Carl Roth, Karlsruhe, Germany), thereby removing fractions of PEI-FITC not bound to the NP surface.

γ-Fe_2O_3-PMA-Dy636

After the polymer coating, the γ-Fe_2O_3-PMA-Dy636 NPs were transferred into sodium borate buffer, and the excess polymer or dye grafted polymer were removed by gel electrophoresis. The protocol was the same as used in previously publications [8, 9]. In this paper, we have used 2% agarose (UltraPure Agarose, Invitrogen) gels and conducted the gel electrophoresis in 0.5 × TBE buffer (44.5 mM Tris-borate and 1 mM EDTA, pH = 8.3, Sigma-Aldrich,) with a potential gradient of 10 V/cm for about 60 min (Figure SI-1.f). To extract the samples from the gels, the separated sample bands were carefully cut from the gels, and then sealed in dialysis membrane tubes with a molecular weight cut off of 3500 kDa (Standard RC Dialysis Tubing, Pre-treated, Spectrum) for about 15 min gel electrophoresis running. Finally the samples were collected from the dialysis membrane tubes and concentrated by ultrafiltration with centrifuge

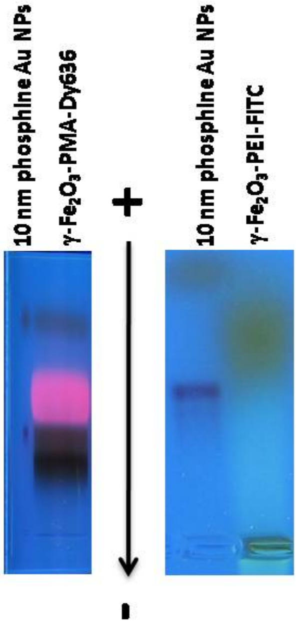

Figure SI-1.f – Purification of γ-Fe_2O_3-PMA-Dy636 NPs by means of gel electrophoresis.
As comparison for stability, γ-Fe_2O_3-PEI-FITC NPs were also run on the gel.

filters (membrane: 100 kDa Mw cut off PES, Sartorius Stedim). γ-Fe_2O_3-PEI-FITC NPs were also submitted to gel electrophoresis and from Figure SI-1.f, it can be seen that the sample γ-Fe_2O_3-PEI-FITC was smeared to a broad band on gel, indicating that they were not resistant to gel electrophoresis and not robust enough compared to γ-Fe_2O_3-PMA-Dy636 NPs. The images of the gels were taken on a ultra-violet light illuminating table. The pink and green colored bands correspond to DY-636 and FITC modified γ-Fe2O3 NPs[3], respectively, and the dark bands above them are due to orange G from the gel loading buffer (30 % glycerol in 0.5× TBE with 0.3 % Orange G) which was mixed with samples by volume ratio of about 1:5. In both gels, the left lane corresponds to 10 nm phosphine coated gold NPs (British Biocell International, #GC10) which acted as a size marker [10]. The arrow denotes the electric field direction.

g) Determination of Fe concentration via ICP-OES

ICP-OES (inductively coupled plasma - optical emission spectroscopy) technology was applied for [Fe] quantification. After lysis of the SPIONs with nitric acid (65 % w/v), the absorbance of the samples was measured at three different wavelengths (238.2 nm, 239.6 nm, 259.9 nm) on an Optima 2000 DV UV/vis

[3]in the case of polymer coated NPs the upper black band corresponds to empty polymer micelles.

absorption spectrometer (PerkinElmer, Rodgau, Germany). Amounts of iron ([Fe]) were calculated by standardization to the internal standard yttrium(III)chloride.

2 Magnetization measurements

The impact of preparation technology on magnetic features of the samples was investigated by monitoring field-dependent magnetization data. Small amounts of lyophilized material (~ 1.5 mg) were placed into a Magnetic Property Measurement System MPMS® equipped with 5 T magnet (Quantum Design, San Diego, CA) using superconducting quantum interference device (SQUID) technology. Magnetization values of the samples were surveyed during a field-dependent sweep from –55,000 to 55,000 Oe at room temperature. All recorded curves showed lack of remanence and typical sigmoidal characteristics. Saturation magnetizations, i.e. the states of maximum alignment of magnetic spins at strong external fields, were derived by tangential fitting of the curve maxima. Numerical values decreased from 54.6 to 23.7 emu g^{-1} for γ-Fe_2O_3-PEI after actual coating with poly(ethylene imine). For counterparts γ-Fe_2O_3-PMA, values were reduced from 27.7 emu g^{-1} for pure γ-Fe_2O_3 after organic synthesis to 16.4 emu g^{-1} (see Table 1 in manuscript)

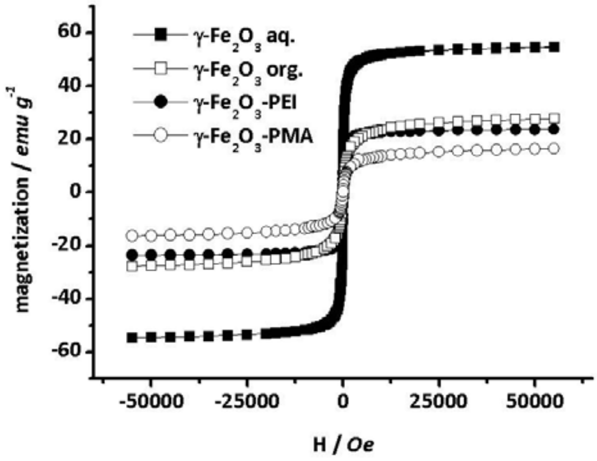

Figure SI-2 – Magnetization of both SPIONs.
The different NPs were measured before (squares) and after (circles) coating.

3 Kinetic of internalization via FACS

The human lung adenocarcinoma cell line A549 was maintained in DMEM high glucose containing 10 % FCS and L-glutamine without the addition of antibiotics in a humidified atmosphere at 37 °C and 8.5 % CO_2. For experiments on uptake kinetics, cells were seeded onto 24-well plates at a density of 60,000 per well. After awaiting adherent growth, culture medium was exchanged with DMEM supplemented with 5 % FCS, in order to avoid interference with the assay reagents. Immediately after this, the wells were incubated with either hydrophilic γ-Fe_2O_3-PEI-FITC (a) or γ-Fe_2O_3-PMA-Dy636 (b) NPs at fixed iron concentrations (1 µg/ml). For the purpose of comparison, blank cells and wells treated with unlabeled NP species were included. Following definite incubation times (15 min, 60 min, 4 h, 24 h), cells were washed twice with PBS, trypsinized and fixed in a 1:1 mixture of FACSFlowTM and paraformaldehyde (4 % w/v). Cell dispersions prepared in this way were analyzed with respect to their fluorescent intensity via flow cytometry (FACS), using a FACSCanto II (BD Biosciences, San Jose, CA). Signals from a total of 10,000 cells were recorded on a detector array system after excitation at 488 nm (channel FITC) and 630 nm (channel APC-A), respectively. Dot plot used for cell gating is shown on the upper left chart. Intracellular iron levels at each time point were quantified by ICP-OES after cell lysis in concentrated nitric acid (600 µl) for 4 hours and subsequent filtration, under the measurement settings described above.

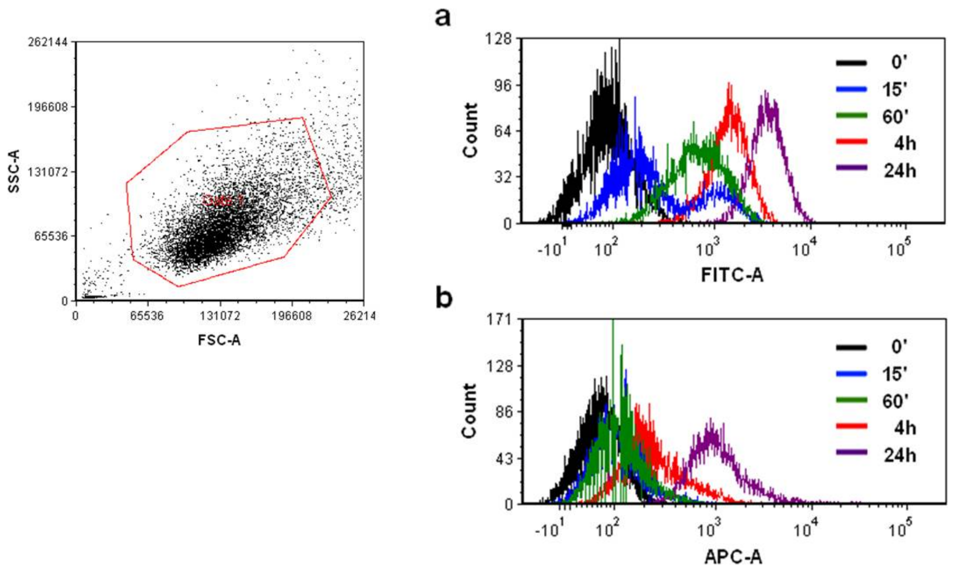

Figure SI-3 – Time-dependent internalization of both SPIONs *via* FACS.

The upper left chart showed the dot plot used for cell gating. On the right chart, the internalization kinetics were measured for (a) γ-Fe_2O_3-PEI-FITC and for (b) γ-Fe_2O_3-PMA-Dy636. The channels used to detect fluorescence signals were FITC-A (488nm) and APC-A (630nm), respectively.

4 Cell culture procedure and characterization via CLSM

Human lung adenocarcinoma cell line A549 was maintained in DMEM high glucose containing 10 % FBS and L-glutamine with the addition of antibiotics (Penicillin/Streptomycin) 37 °C and 5 % CO_2. For experiments on uptake kinetics, cells were seeded onto 24-well plates at a density of 32,000 cells/cm². After awaiting adherent growth, culture medium was exchanged with DMEM supplemented with 5 % FBS, in order to decrease agglomeration of the positively charged NPs. Immediately after this, the wells were incubated with either γ-Fe_2O_3-PEI-FITC or γ-Fe_2O_3-PMA-Dy636 NPs or both NP species simultaneously at fixed iron concentrations (1 µg/ml) for 15 min, 30 min, 60 min, 2 h, 4 h, 8 h and 24 h (depending on the experiments). For the purpose of comparison, blank cells and wells treated with unlabeled particle species were included. For examination a CLSM 510 Meta (Zeiss) microscope was used equipped with a laser diode emitting at 405 nm, an argon laser with a line at 488 nm, a helium-neon laser for excitation at 543 nm and an additional helium-neon laser emitting at 633 nm.

5 Quantification of intracellular iron concentration

As intensity signals were measured either on different FACS channels or in the CLSM and labeling efficiencies of the two NP systems were not homogenous, absolute comparability between γ-Fe_2O_3-PEI-FITC and γ-Fe_2O_3-PMA-Dy636 NPs was not given before quantification of cell iron following analogous incubation regimes (red bars). When cells were exposed to γ-Fe_2O_3-PEI-FITC NPs at an initial dose of 1 µg [Fe], about 12 % of these NPs was found to be internalized after 24 hours. Consistent with the findings from flow cytometry, the uptake of this species increased constantly over time. Unlike that, γ-Fe_2O_3-PMA-Dy636 NPs invaded A549 cells to a significantly lower amount of 1.5 %. Data points after 15 and 60 minutes even lay within the measurement inaccuracy, and the most articulate rise in intracellular iron mass appeared after 4 h. Nevertheless, the results from ICP-OES correlated well with the mean fluorescence intensity (MFI) values from flow cytometry (green bars) as indicated by the calculated coefficients of determination for each of the formulations (R^2 = 0.993 and R^2 = 0.990 for γ-Fe_2O_3-PEI-FITC and γ-Fe_2O_3-PMA-Dy636 NPs, respectively). It has to be pointed out that despite an optimization of the cell culture media and NP dose (*i.e.* reduced serum quantity as well as adequate NP dose) to increase the stability of the γ-Fe_2O_3-PEI-FITC NPs, aggregation was still observed for this formulation and the agglomerates were strongly attached to the cell membrane (Figure SI-6.a.iii.). Trypsinization of the cells did not remove the agglomerates of γ-Fe_2O_3-PEI-FITC NPs attached to the cell membrane so that the results obtained for the iron quantification were affected with a high probability. In this regard, a

contribution of the extracellularly located γ-Fe_2O_3-PEI-FITC NPs to the increased signal obtained by flow cytometry and by ICP-OES cannot be neglected.

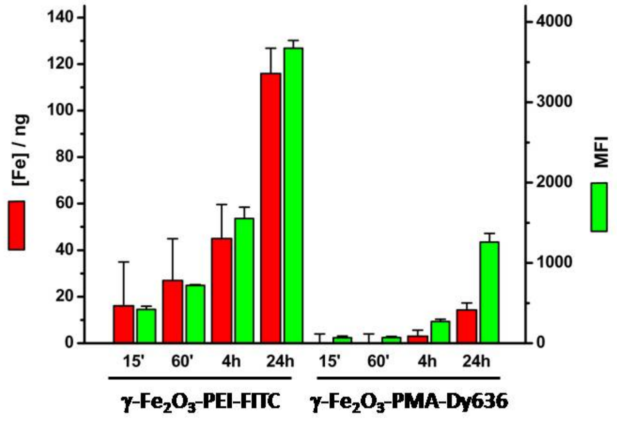

Figure SI-5 – Quantification of intracellular iron concentration for both SPIONs upon time.

6 Kinetic of internalization and intracellular localization of SPIONs via CLSM: concentration and time dependence

a. Concentration dependence

A549 cells were treated with the different SPIONs (γ-Fe_2O_3-PEI-FITC and γ-Fe_2O_3-PMA-Dy636 NPs) at the concentrations indicated for 24 h (Fig. SI-6.a.i). Afterwards the cells were trypsinized and intensively washed to remove possible agglomerates and seeded again in a freshly media. To confirm intracellular localization the cell membrane and the nucleus were stained with 60 µg/ml WGA-Alexa (594 or 488, respectively) and DAPI (0.4 µM). Due to the poor colloidal stability of the γ-Fe_2O_3-PEI-FITC NPs (*cf.* Fig. SI-1.f) in the cell media (Fig SI-6.a.iii), big agglomerates were formed at concentrations higher than of 1 µg/ml. At 10 µg/ml, the viability of the cells was clearly affected (Fig. SI-6.a.ii) so that no picture could be used for analysis. This was one of the reasons taken into account to establish the final incubation dose at 1 µg/ml.

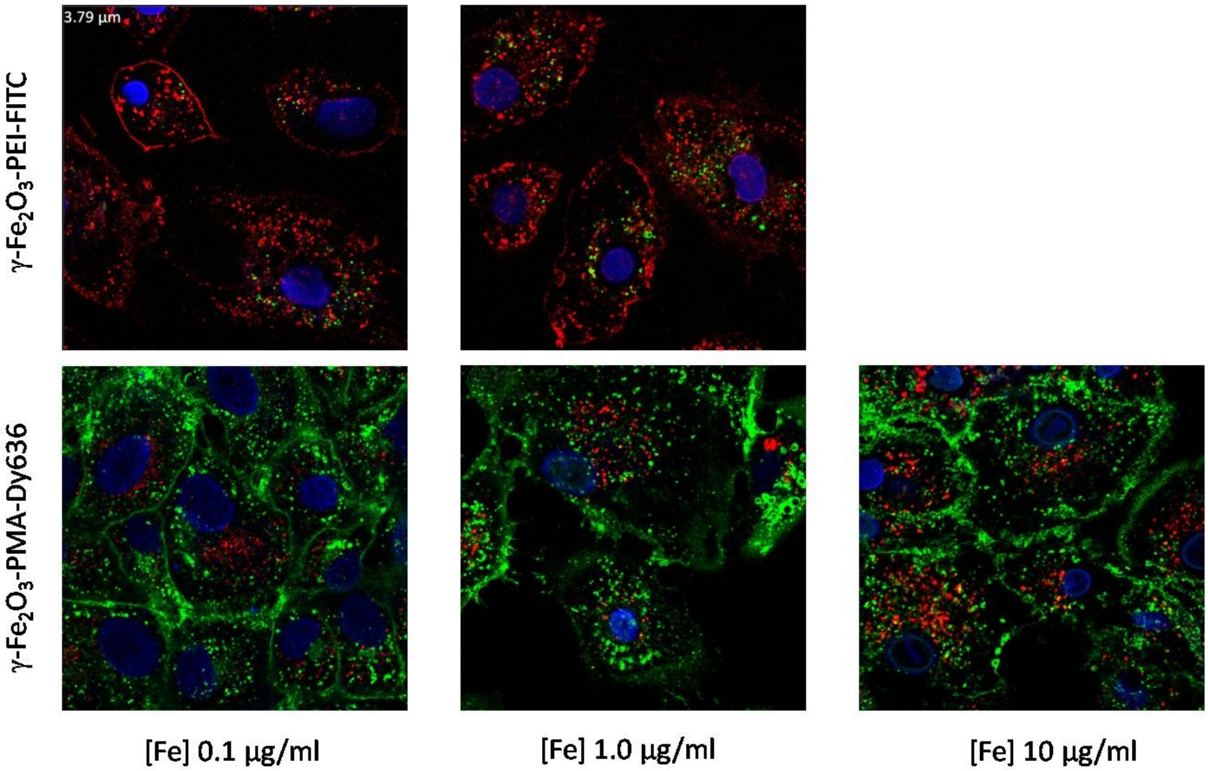

Figure SI-6.a.i - Concentration-dependent internalization of both SPIONs *via* CLSM.

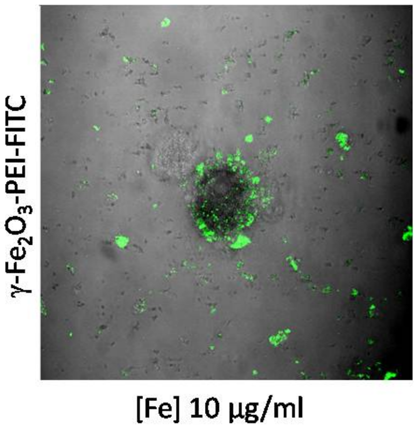

Figure SI-6.a.ii – Agglomeration of γ-Fe_2O_3-PEI-FITC (green) at high concentration in cell culture medium.

In the picture below, Figure SI-6.a.iii, it can be clearly seen that positively charged γ-Fe_2O_3-PEI-FITC NPs are rather unstable in media. Despite optimization of cell culture conditions and the time/dose of incubation, strong agglomerates are found on the outer side of the plasma membrane of A549 cells as demonstrated by the colocalization of the green γ-Fe_2O_3-PEI-FITC NPs with the red stained cellular membrane. Trypsinization of the cells was ineffective removing agglomerates and made impossible any kind of analysis in live cells. Therefore, for the rest of experiments the cells were fixed for immunostaining after the desired incubation time. Due to the different steps within this procedure, the membrane is strongly damaged so that possible agglomerates were effectively removed.

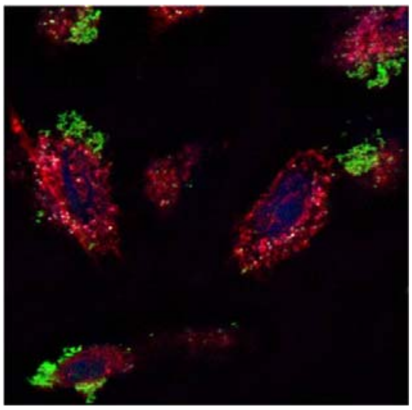

Figure SI-6.a.iii – Attachment of agglomerated γ-Fe_2O_3-PEI-FITC to the plasma membrane of the cells. Cells were incubated with the positively charged NPs (green) at 1 µg/ml iron concentration and stained for CLSM analysis. The plasma membrane was stained with wheat-germ agglutinin-Alexa Fluor 546 (red) and the nucleus with DAPI (blue).

b. Time dependence and intracellular localization of both SPIONs

For the fluorescence staining of cell membranes, actin, nuclei, endosomes, and lysosomes, A549 adenocarcinoma cells were treated as described in §**Error! Reference source not found.**, except the fact, that they were grown on coverslips of 12 mm diameter. In detail, the cells were washed twice with PBS previous fixation with paraformaldehyde (8% w/v, Electron Microscopy Sciences) diluted with PBS 1:1 for 30 min at RT. In the next step either the plasma membrane (i) of the cells or the actin cytoskeleton (ii) was stained. In (i) triple washing that followed was performed with HBSS to equilibrate cells for plasma membrane staining. Wheat germ agglutinin-tetramethylrhodamine (Invitrogen) was applied at a concentration of 40 µg/ml for 20min and the cells were washed twice with HBSS and once with PBS afterwards. In (ii) the cells were washed three times with PBS instead and were treated with phalloidin-tetramethylrhodamine (Sigma) at 200 nM diluted in PBS followed by triple washing with PBS. In both cases the cells were afterwards permeabilized with 50 mg Glycin and 5 mg Saponin dissolved in 10 ml

PBS for 5 min. Then, cells were washed with PBS and incubated in blocking solution (140 mg BSA dissolved in 7 ml permeabilization solution). Primary antibodies were diluted in blocking solution as follows and applied to cells for 1 h at 37 °C. The monoclonal mouse anti-human LAMP1/CD107a antibodies (Developmental Studies Hybridoma Bank) was used at a final concentration of 1 µg/ml and the stock solution of the polyclonal rabbit anti-human EEA1 immunoglobulin (Cell Signaling) was diluted 1:50. Afterwards, the cells were washed three times with PBS containing 1% BSA and exposed to secondary fluorescent labeled antibodies. The donkey anti-mouse DyLight405 antibody (Jackson ImmunoResearch) was used at 1 µg/ml to detect the LAMP1 specific primary antibodies while the goat anti-rabbit AlexaFlour430 conjugated immunoglobulin (Invitrogen) was used as secondary antibodies for early endosomes at 30 µg/ml. Finally, the cells were washed three times with PBS and mounted on slides using Flouromount-G (Southern Biotech). The concentrations of antibodies, wheat germ agglutinin-tetramethylrhodamine and phalloidin-tetramethylrhodamine respectively were determined very carefully in additional experiments to minimize any crosstalk with fluorescent signals coming from the NP systems. Crosstalk signals were prejudicial for the analysis of colocalization quantitatively but also qualitatively. Even a minimal interference led to wrong results during the quantification of the colocalization. Furthermore, to be linearly unmixed the corresponding fluorescent images have to be acquired after excitation with the same wavelength. For example, when illuminating a probe at 488 nm not only γ-Fe_2O_3-PEI-FITC NPs but also the secondary antibody against the early endosomal structures (AlexaFlour 430), was slightly excited. It turned out, that typical gain settings of the photomultiplier and the laser power used to excite γ-Fe_2O_3-PEI-FITC NPs were too low to detect any signal from a control with cells containing no traces of NPs but were stained for endosomes. An equilibrium between antibody-labeling and microscope settings war crucial to obtain reliable results regarding the colocalization of the NPs with the different organelles.

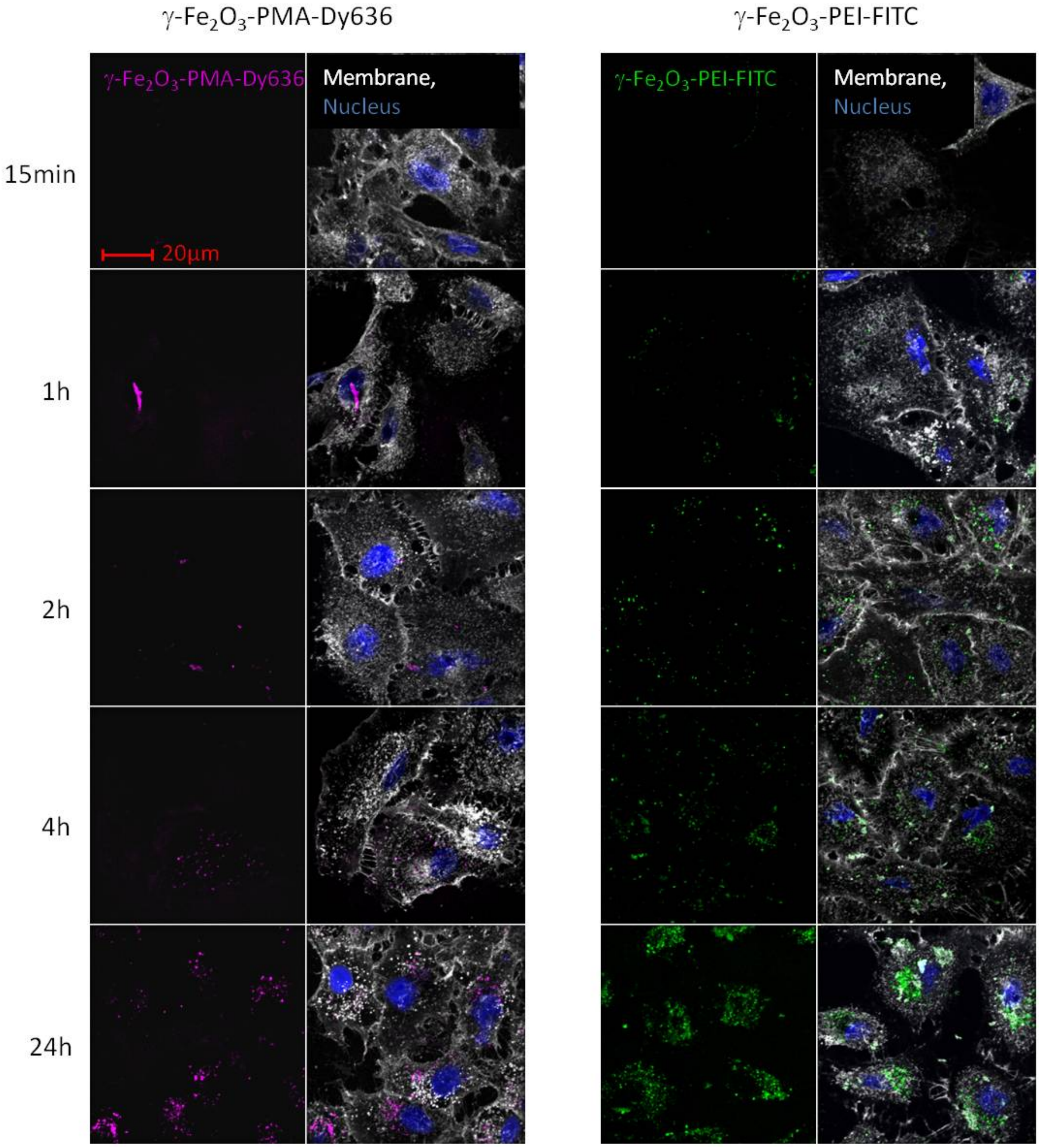
γ-Fe_2O_3-PMA-Dy636
γ-Fe_2O_3-PEI-FITC
γ-Fe_2O_3-PMA-Dy636
Membrane,
Nucleus
γ-Fe_2O_3-PEI-FITC
Membrane,
Nucleus
15min
1h
2h
4h
24h
20μm

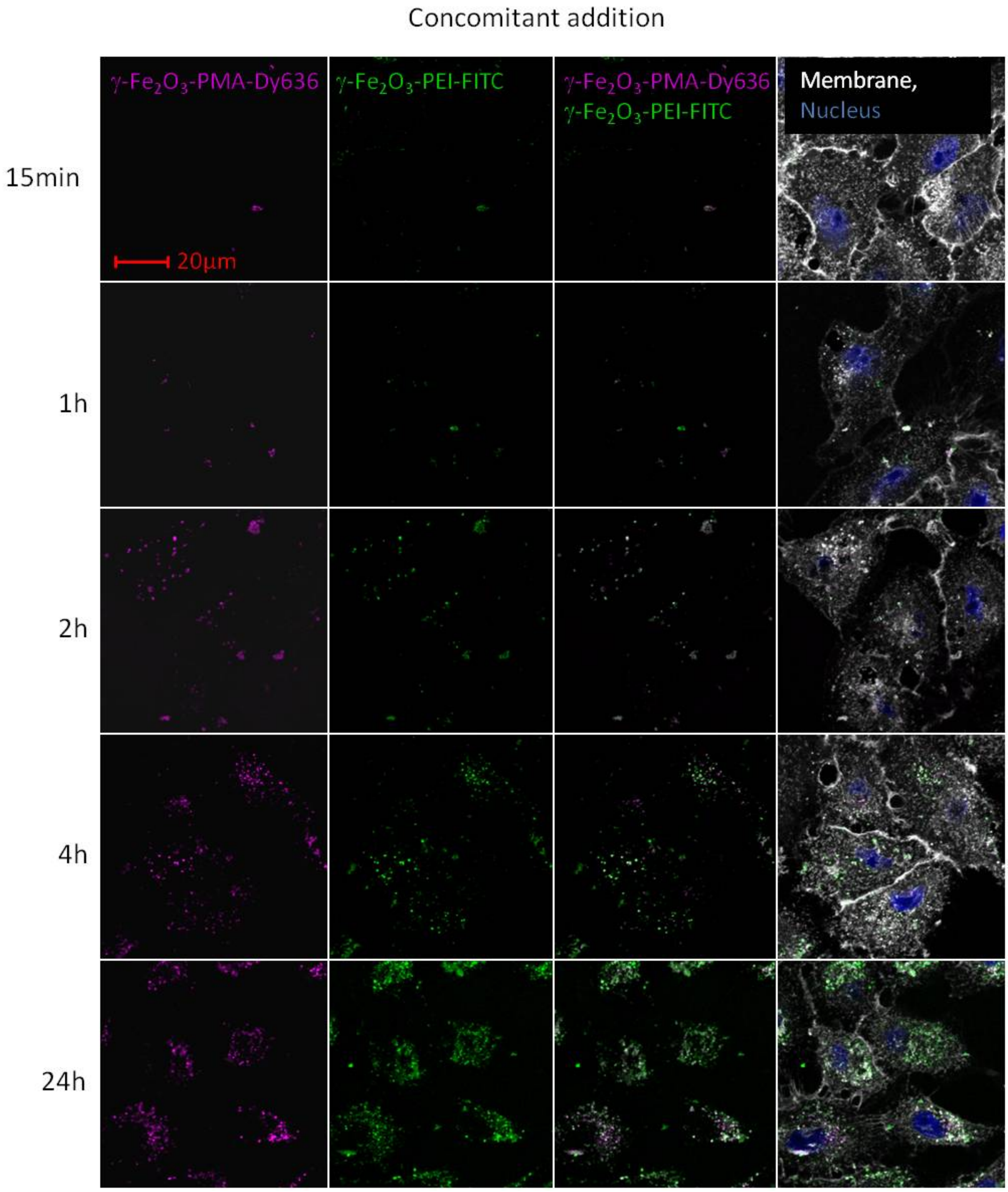
Concomitant addition
γ-Fe2O3-PMA-Dy636
γ-Fe2O3-PEI-FITC
γ-Fe2O3-PMA-Dy636
γ-Fe2O3-PEI-FITC
Membrane,
Nucleus
15min
1h
2h
4h
24h
20μm

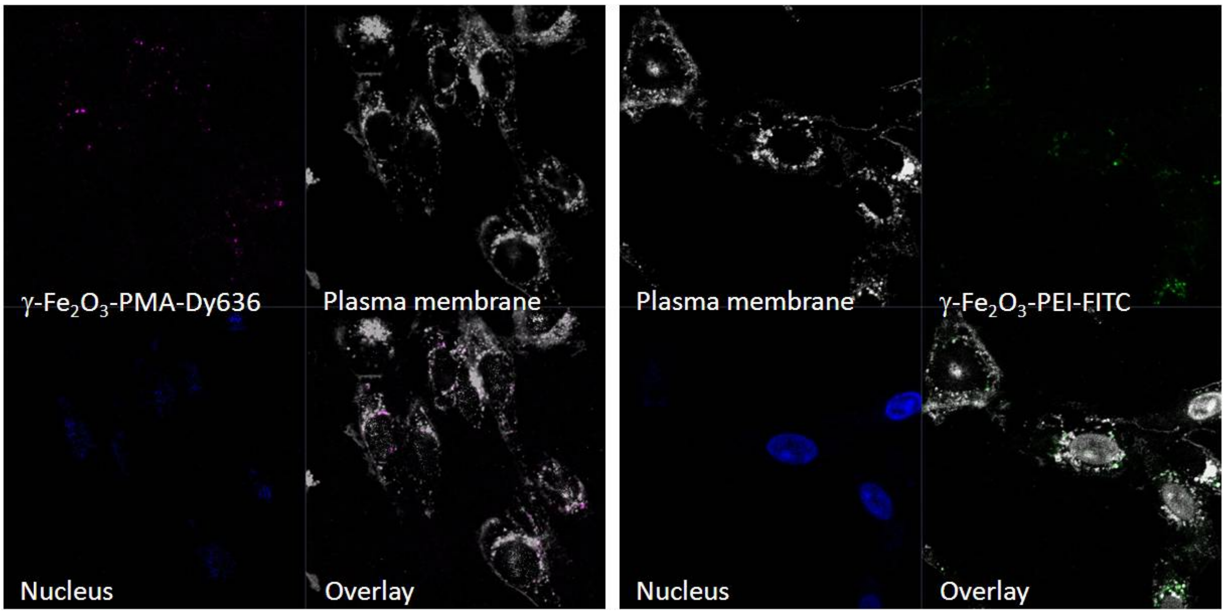

Figure SI-1.b.i – Time dependent internalization of both SPIONs.

Cells were incubated for the indicated times either with single γ-Fe_2O_3-PEI-FITC (green) or γ-Fe_2O_3-PMA-Dy636 (rosa) NPs (upper chart) or both NPs concomitantly (middle chart). In agreement with the results obtained by FACS, positively charged NPs were faster internalized than the negatively charged counterparts. The peak of internalization observed by CLSM was 2 h and 8 h (low chart) for the γ-Fe_2O_3-PEI-FITC (green) and γ-Fe_2O_3-PMA-Dy636 NPs, respectively. Interestingly, when both systems were added simultaneously to the cells, the kinetic of internalization of the negatively charged NPs was significantly increased. Thus, γ-Fe_2O_3-PMA-Dy636 NPs were clearly visible inside the cells after 4 h.

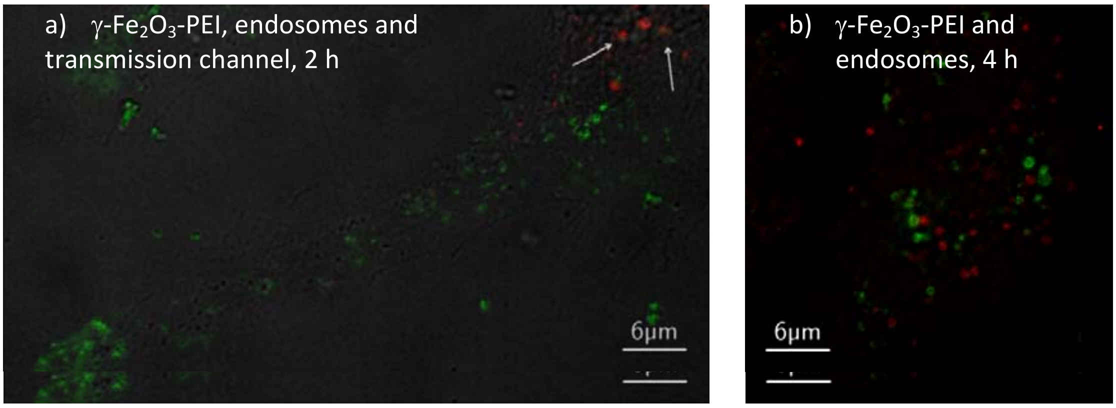

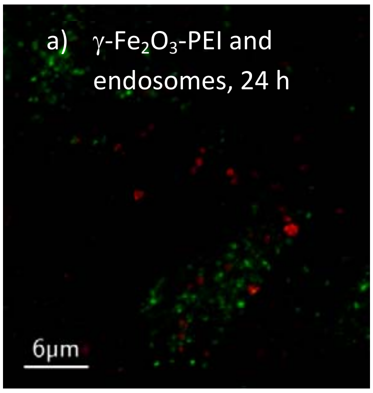

Figure SI-2.b.ii – Colocalization of γ-Fe_2O_3-PEI-FITC NPs with the endosomes

Endosomes: red, NPs: green. For t = 2 h (a) only a low amount of γ-Fe_2O_3-PEI-FITC NPs can be found inside endosomes (arrows), the major fraction is sticking to the plasma membrane. γ-Fe_2O_3-PEI-FITC NPs are neither colocalizing with the endosomes after 4 h (b), nor after 24 h (c) of incubation time.

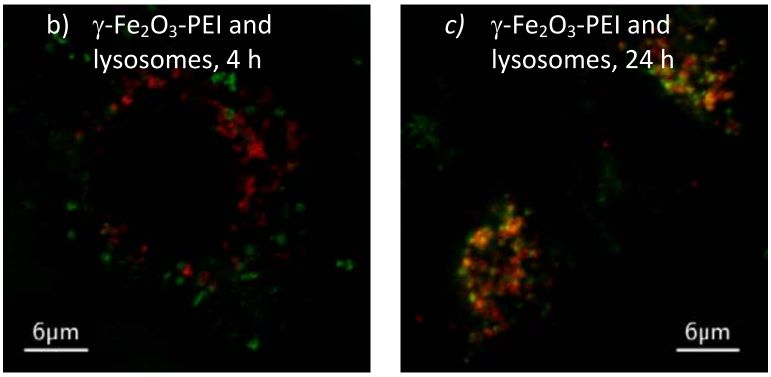

Figure SI-6.b.iii – Colocalization of γ-Fe_2O_3-PEI-FITC NPs with the lysosomes

Lysosomes: red, NPs: green. γ-Fe_2O_3-PEI-FITC are not present within lysosomal structures after 4 h (a). After 24 h enrichment in these structures is clearly visible (b).

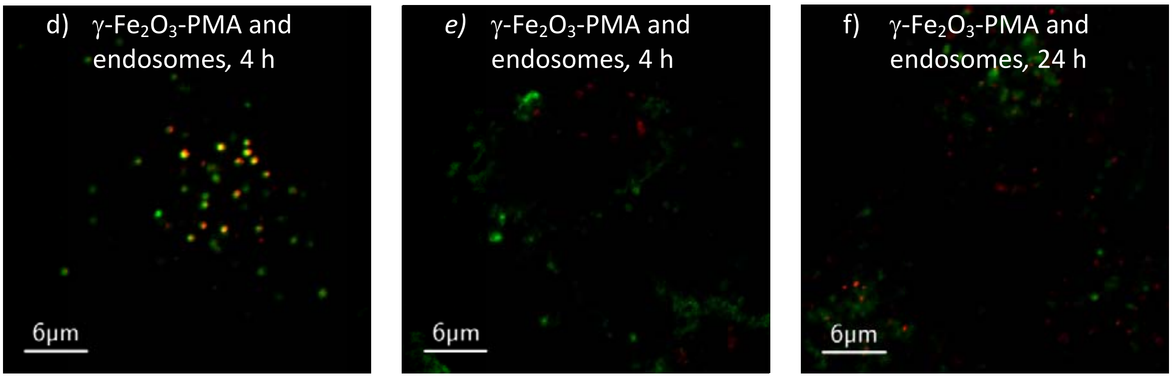

Figure SI-6.b.iv – Colocalization of γ-Fe_2O_3-PMA-Dy636 NPs with the endosomes

Endosomes: red, NPs: green. After 4 h γ-Fe_2O_3-PMA-Dy636 particles can be found inside endosomes within a fraction of the cell population (a), in other selected cells no overlap is visible (b). In the majority of analyzed cells no fluorescence signal of PMA-coated NPs was detectable after 4 h. After 24 h particles colocalize only partially with endosomes (c).

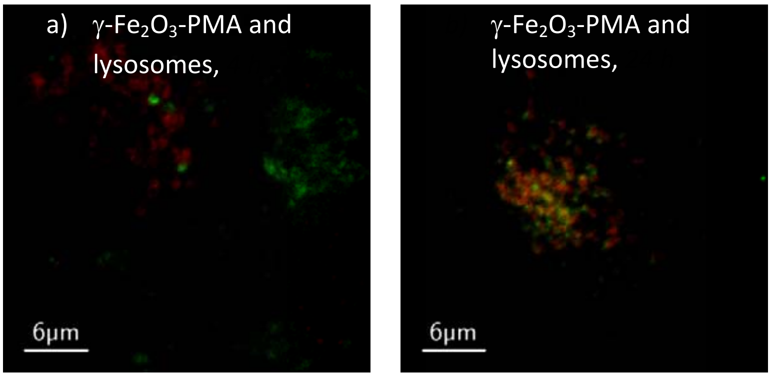

Figure SI-6.b.v - Colocalization of γ-Fe_2O_3-PMA-Dy636 NPs with the lysosomes

Lysosomes: red, NPs: green. After 4 h no colocalization between γ-Fe_2O_3-PMA-Dy636 particles and lysosomes is visible (a). After 24 h the fluorescence signal of NPs and stained lysosomes is overlapping.

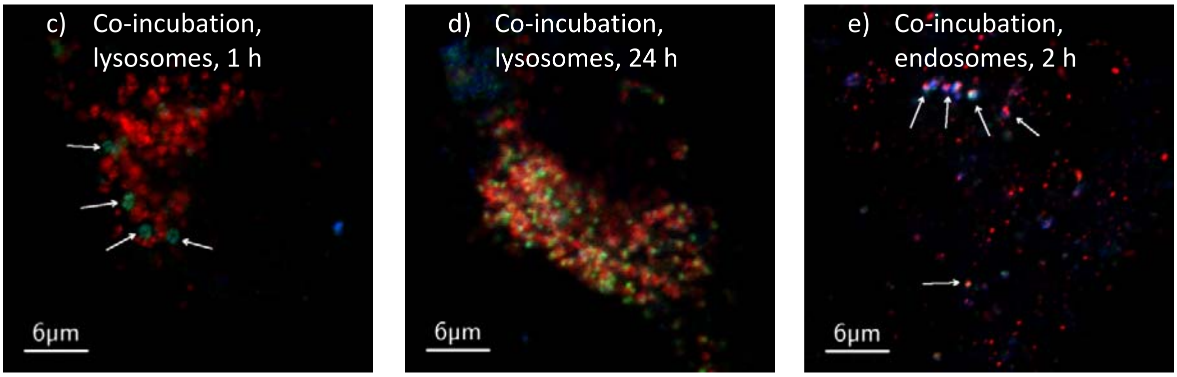

Figure SI-6.b.vi – Internalization of γ-Fe_2O_3-PEI-FITC and γ-Fe_2O_3-PMA-Dy636 NPs (co-incubation)

Lysosomes: red, γ-Fe_2O_3-PEI-FITC NPs: blue, γ-Fe_2O_3-PMA-Dy636 NPs: green. After 1 h incubation time both nanoparticle systems do not colocalize wth lysosomes (a). After 24 h both particles are present in lysosomalen structures (b). In (c) the colocalization between endosomes and both nanoparticle species after 3 h of incubation time is visible.

The polycation PEI, which is used as a surface coating for the positively charged NPs, is known as a membrane destabilizing agent [11]. In some cases green fluorescence of PEI coated NPs could be detected outside of endosomes/lysosomes within the cytosol which might indicate permeabilization of intracellular membranes due to the effect of PEI (Fig. SI-6.b.vii). Therefore, the localization of the NPs with respect to the cytosol was also studied. For this purpose, the actin cytoskeleton was stained with phalloidin-tetramethylrhodamine (shown in blue) as described before while traces of γ-Fe_2O_3-PEI-FITC NPs are shown in green. As can be seen in the Figure below (Figure SI-6.c), any sign of cytosolic γ-Fe_2O_3-PEI-FITC NP distribution could be concluded.

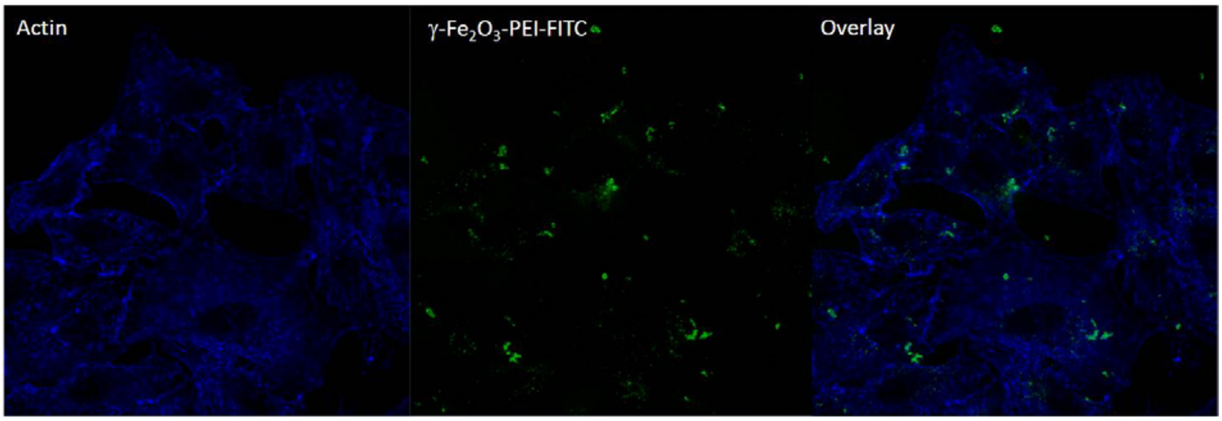

Figure SI-6.c – Colocalization of γ-Fe_2O_3-PEI-FITC NPs with the cytosol.

Actin cytoskeleton: blue, γ-Fe_2O_3-PEI-FITC NPs: green. After 8 h incubation there were any signs of cytosolic distribution of the NPs.

7 Quantitative analysis of colocalization studies

A549 adenocarcinoma cells were treated as described in §**Error! Reference source not found.**, except the fact, that they were grown on coverslips of 12 mm diameter. For the intensive quantitative colocalization studies the intracellular locations of early endosomes and lysosomes with both nanoparticle species were correlated for each of the given incubations times as shown in

Table 1. Therefore either endosomes or lysosomes were stained as described in §6. For each combination at least 20 cells were imaged using a highly corrected CLSM 510 Meta (Zeiss).

Nanoparticle species	*Organelle*
γ-Fe_2O_3-PEI	early endosomes
γ-Fe_2O_3-PEI	lysosomes
γ-Fe_2O_3-PMA	early endosomes
γ-Fe_2O_3-PMA	lysosomes
γ-Fe_2O_3-PEI + γ-Fe_2O_3-PMA	early endosomes
γ-Fe_2O_3-PEI + γ-Fe_2O_3-PMA	lysosomes
γ-Fe_2O_3-PEI	γ-Fe_2O_3-PMA

Table 1 - Correlation experiments

The image material was corrected for noise by median filtering and thresholding. [12] To quantify the degree of colocalization between fluorescence signal originating from NPs and labeled endosomes (EEA1) or lysosomes (LAMP1) various correlation coefficients given in Table 2 were calculated based on confocal

images. Manders' distinct colocalization coefficients M_1 and M_2 [13] were chosen for further interpretation:

$$M_1 = \frac{\sum R_{i,coloc}}{\sum R_i} \in [0,1] \text{ and } M_2 = \frac{\sum G_{i,coloc}}{\sum G_i} \in [0,1] \qquad (1)$$

R_i and G_i are the pixel intensities of pixel i in channel R and G. "*coloc*" are pixels in which colocalization was observed. Considering a dual-color confocal image with two components, *i.e.* fluorescence signal from NPs and stained intracellular organelles, the numbers of objects in the two components of the image are not equal. Therefore the calculation of one single correlation coefficient does not cover the situation, where most of signal originating from NPs is co-localizing with intracellular organelles though the highest fraction of the organelles is not filled with particles for example. In our calculations M_1 represents the degree of colocalization of fluorescence signal from one nanoparticle species with signal either coming from stained endosomes or lysosomes while M_2 covers the situation with regard to the organelles.

Example	R_r	***R***	M_1	M_2	***ICQ***
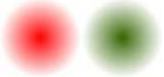	*-0,51*	*0*	*0*	*0*	*0,06*
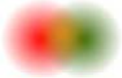	*-0,37*	*0,19*	*0,36*	*0,36*	*0,02*
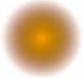	*1,00*	*1,00*	*1,00*	*1,00*	*0,50*
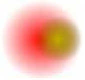	*0,37*	*0,58*	*0,46*	*1,00*	*0,15*

Table 2 – Correlation coefficients

R_r: Pearson's correlation coefficient [14] R: Manders' overlap coefficient [13], M_1 andM_2: Manders' distinct colocalization coefficients [13], ICQ: Intensity correlation coefficient [15]

Results

The degree of correlation between positively charged PEI-coated NPs and early endosomes after various incubation times is shown in the left part of Figure SI-7. Manders' colocalization coefficients are close to zero for both structures (PEI-coated NPs: green, EEA1: yellow). The lack of signal overlap is also visible in Figure SI-3.b.ii exemplary. Nevertheless Pearson's correlation coefficient is slightly higher for short incubations times (blue). After 8 h a high amount of NPs can be found inside the lysosomes (right part of Figure SI-7.i, PEI-coated NPs: green, LAMP1: red) but the major fraction of lysosomal structures still does not contain any particles. For t=24 h both coefficients converge. Confocal image data underlining these observations is provided in Figure SI-6.

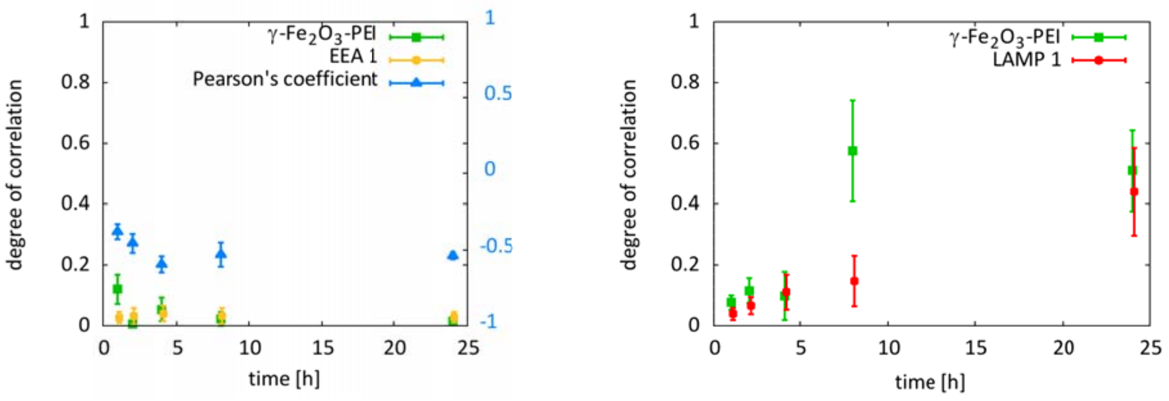

Figure SI-7.i - Internalization of γ-Fe_2O_3-PEI-FITC NPs
Shown are Manders' distinct colocalization coefficients (M_1: green, M_2: yellow/red) and Pearson's correlation coefficient (blue).

The degree of correlation between positively charged PMA-coated particles and early endosomes is shown in the left part of Figure SI-7.ii (PMA-coated NPs: magenta, EEA1: yellow). The colocalization between γ-Fe_2O_3-PMA-Dy636 NPs and endosomes is highest for t = 4 h. Large error bars indicates that the uptake is very inhomogeneous. The enrichment of γ-Fe_2O_3-PMA-Dy636 NPs inside lysosomes is visible after 24 h (Error! Reference source not found. SI-7.ii, right). See Figure SI-6 for qualitative results.

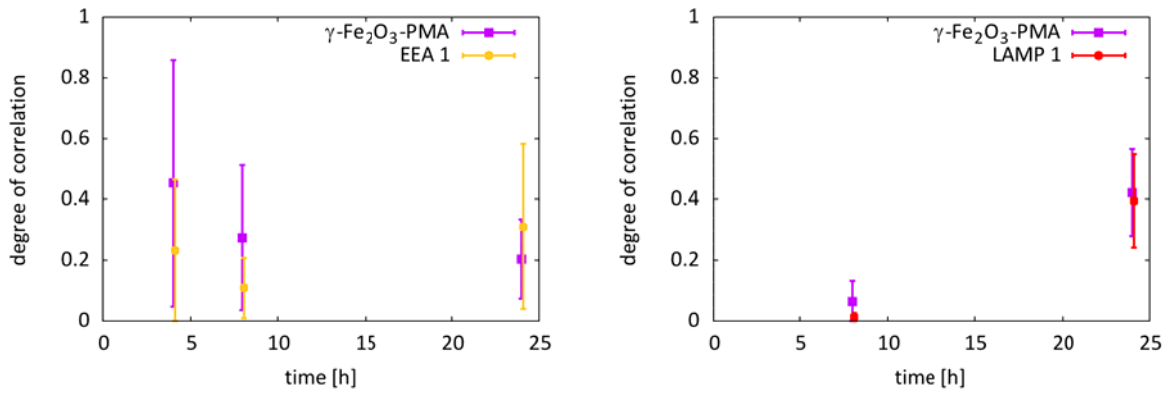

Figure SI-7.ii - Internalization of γ-Fe_2O_3-PMA-Dy636 NPs
Shown are Manders' distinct colocalization coefficients (M_1: magenta, M_2: yellow/red).

If the cells are exposed to both NPs systems simultaneously the intracellular distribution is totally different: After 4 h the value of the coefficient representing the colocalization of γ-Fe_2O_3-PEI-FITC NPs with early endosomes has a maximum (Figure SI-7.iii, left, green). Afterwards it is decreasing again. The EEA1-coefficient is highest for t = 8 h and stays at a high level for t > 8 h (yellow). The fraction of PEI-NPs enriched in lysosomal structures is increasing steadily (Figure SI-7.iii, right).

After 2 h the value of the coefficient representing the colocalization of γ-Fe_2O_3-PMA-Dy636 NPs with early endosomes has a maximum (Figure SI-7.iv, left, magenta). Afterwards it is decreasing again. The EEA1-coefficient is highest for t = 8 h and decreases slightly afterwards (yellow). The fraction of NPs enriched in lysosomal structures is increasing steadily (Figure SI-7.iv, right). Comparing this Figure with Figure SI-7.iii during co-incubation the intracellular transport of γ-Fe_2O_3-PMA-Dy636 NPs towards the endosomes takes place faster than those of γ-Fe_2O_3-PEI-FITC NPs.

For confocal images covering these cases exemplary refer to Figure SI-6.

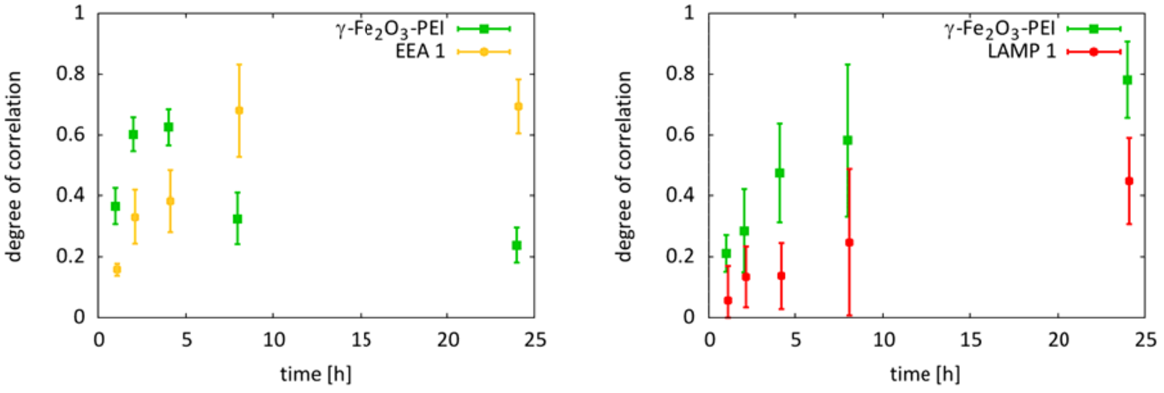

Figure SI-7.iii – Co-incubation: Internalization of γ-Fe_2O_3-PEI-FITC NPs
Shown are Manders' distinct colocalization coefficients (M_1: green, M_2: yellow/red).

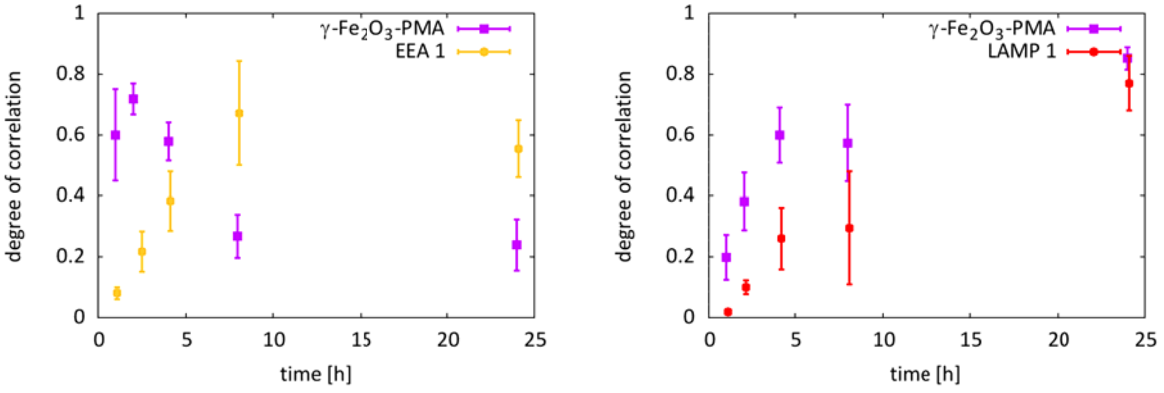

Figure SI-7.iv – Co-incubation: Internalization of γ-Fe_2O_3-PMA-Dy636 NPs
Shown are Manders' distinct colocalization coefficients (M_1: magenta, M_2: yellow/red).

Finally the colocalization between both nanoparticle species after co-incubation was evaluated by calculating the ICQ values and Manders' overlap-coefficient as shown in Figure SI-7.v. The uptake pattern

differs for t < 4 h. This might be caused by the earlier detection of γ-Fe_2O_3-PMA-Dy636 NPs inside endosomal structures in comparison to γ-Fe_2O_3-PEI-FITC NPs (compare left parts of Figure SI-7.iii and Figure SI-7.iv).

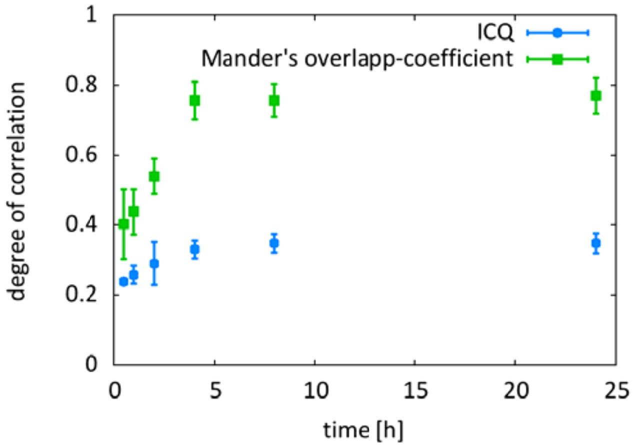

Figure SI-7.v – Co-incubation: Spatial correlation between fluorescence signal from γ-Fe_2O_3-PEI-FITC and γ-Fe_2O_3-PMA-Dy636 NPs.
Shown are the intensity correlation coefficient (blue) and Manders' overlap coefficient (green).

8 References

1. Bee A, Massart R, Neveu S: **Synthesis of Very Fine Maghemite Particles**. *Journal of Magnetism and Magnetic Materials* 1995, **149**:6-9.
2. Hyeon T: **Chemical synthesis of magnetic nanoparticles**. *Chem Commun* 2003, 8:927-934.
3. Gautam A, Densmore CL, Golunski E, Xu B, Waldrep JC: **Transgene expression in mouse airway epithelium by aerosol gene therapy with PEI-DNA complexes**. *Molecular Therapy* 2001, **3**:551-556.
4. Pellegrino T, Manna L, Kudera S, Liedl T, Koktysh D, Rogach AL, Keller S, Rädler J, Natile G, Parak WJ: **Hydrophobic nanocrystals coated with an amphiphilic polymer shell: a general route to water soluble nanocrystals**. *Nano Letters* 2004, **4**:703-707.
5. Sperling RA, Pellegrino T, Li JK, Chang WH, Parak WJ: **Electrophoretic separation of nanoparticles with a discrete number of functional groups**. *Advanced Functional Materials* 2006, **16**:943-948.
6. Fernández-Argüelles MT, Yakovlev A, Sperling RA, Luccardini C, Gaillard S, Medel AS, Mallet J-M, Brochon J-C, Feltz A, Oheim M, Parak WJ: **Synthesis and characterization of polymer-coated quantum dots with integrated acceptor dyes as FRET-based nanoprobes**. *NanoLetters* 2007, **7**:2613-2617.
7. Lin C-AJ, Sperling RA, Li JK, Yang T-Y, Li P-Y, Zanella M, Chang WH, Parak WJ: **Design of an amphiphilic polymer for nanoparticle coating and functionalization**. *Small* 2008, **4**:334-341.
8. Yakovlev AV, Zhang F, Zulqurnain A, Azhar-Zahoor A, Luccardini C, Gaillard S, Mallet JM, Tauc P, Brochon JC, Parak WJ, et al: **Wrapping Nanocrystals with an Amphiphilic Polymer Preloaded with Fixed Amounts of Fluorophore Generates FRET-Based Nanoprobes with a Controlled Donor/Acceptor Ratio**. *Langmuir* 2009, **25**:3232-3239.

9. Zhang F, Ali Z, Amin F, Feltz A, Oheim M, Parak WJ: **Ion and pH Sensing with Colloidal Nanoparticles: Influence of Surface Charge on Sensing and Colloidal Properties**. *ChemPhysChem* 2010, **11**:730-735.
10. Pellegrino T, Sperling RA, Alivisatos AP, Parak WJ: **Gel electrophoresis of Gold-DNA Nanoconjugates**. *Journal of Biomedicine and Biotechnology* 2007, article ID 26796: DOI: 10.1155/2007/26796.
11. Creusat G, Rinaldi AS, Weiss E, Elbaghdadi R, Remy JS, Mulherkar R, Zuber G: **Proton sponge trick for pH-sensitive disassembly of polyethylenimine-based siRNA delivery systems**. *Bioconjug Chem* 2010, **21**:994-1002.
12. Landmann L, Marbet P: **Colocalization analysis yields superior results after image restoration**. *Microscopy Research And Technique* 2004, **64**:103-112.
13. Manders EMM, Verbeek FJ, Aten JA: **Measurement Of Colocalization Of Objects In Dual-Color Confocal Images**. *Journal Of Microscopy-Oxford* 1993, **169**:375-382.
14. Gonzalez RC, Woods RE: **Digital Image Processing**. 2008.
15. Li Q, Lau A, Morris TJ, Guo L, Fordyce CB, Stanley EF: **A Syntaxin 1, Galpha(o), and N-Type Calcium Channel Complex at a Presynaptic Nerve Terminal: Analysis by Quantitative Immunocolocalization**. *The Journal of Neuroscience* 2004, **24**:4070-4081.
